# Endothelial c-IAP2 loss amplifies P2X7 receptor-driven inflammation and worsens schistosomiasis-associated pulmonary hypertension

**DOI:** 10.1073/pnas.2513158123

**Published:** 2026-06-15

**Authors:** Elizabeth Sue Villarreal, Ygor Marinho, Omar Loya, Samuel Yaw Aboagye, Gabriela Costa, Fernanda Oliveira, Muskan Gupta, Katia Costa Oliveira, Claudia Lucia Martins Silva, Jun Sun, Serpil Cem Erzurum, Sarah Elizabeth Lutz, David Lee Williams, Rudolf Krawczenko Feitoza Oliveira, Vinicio de Jesus Perez, Suellen Darc Oliveira

**Affiliations:** ^a^ Vascular Immunobiology Lab, College of Medicine, Department of Anesthesiology, University of Illinois Chicago, Chicago, IL 60612; ^b^ Department of Microbial Pathogens and Immunity, Rush University, Chicago, IL 60612; ^c^ Department of Medicine, Federal University of Sao Paulo, Sao Paulo, SP 04023-062, Brazil; ^d^ Department of Anatomy and Cell Biology, University of Illinois Chicago, Chicago, IL 60612; ^e^ Department of Microbiology, Immunology, and Parasitology, Federal University of Sao Paulo, Sao Paulo, SP 04023-062, Brazil; ^f^ Program of Pharmacology and Medicinal Biochemistry, Federal University of Rio de Janeiro, Rio de Janeiro, RJ 21941-902, Brazil; ^g^ College of Medicine, Department of Medicine, University of Illinois Chicago, Chicago, IL 60612; ^h^ Lerner Research Institute, Cleveland Clinic Foundation, Cleveland, OH 44195; ^i^ Division of Pulmonary and Critical Care, Stanford University, Palo Alto, CA 94305; ^j^ Department of Physiology and Biophysics, University of Illinois Chicago, Chicago, IL 60612

**Keywords:** PAH, endothelial cells, apoptosis, P2X7R, c-IAP2

## Abstract

Schistosomiasis-associated pulmonary hypertension (Sch-PH) is the most common form of group I PH worldwide. Recently, data revealed that the preclinical Sch-PH animal model exhibited gut and lung microbiome dysbiosis, associated with significant lung endothelial cell (EC) dysfunction and microvascular apoptosis. However, the role of pro-/antiapoptosis sensors, such as the inhibitor of apoptosis protein 2 (c-IAP2) and the purinergic receptor P2X7 (P2X7R), remained unclear. Using Cdh5cre-ER^T2^;cIAP1^−/−^;cIAP2^fl/fl^ animal model, this study investigated the contribution of endothelial c-IAP2 in this process, revealing pulmonary P2X7R overexpression as a putative target in the onset of Sch-PH. Pharmacologically, inhibition of P2X7R function confirmed its role in promoting lung EC death and disease progression. Moreover, data suggest that microbiome-associated metabolic alterations in Sch-PH seem linked to microvascular EC apoptosis driven by ATP/P2X7R overactivation and suppressed c-IAP2 expression. Indeed, genetic ablation of endothelial c-IAP2 expression was sufficient to induce PH-like features in mice, with echocardiography indicating a higher pulmonary acceleration time (PAT), PAT/pulmonary ejection time, and right ventricular free wall thickness (RVFWTH) after IP/IV-Egg challenge compared to controls, an effect linked to the female prevalence of the disease. These findings suggest a significant contribution of lung EC-P2X7R activation and c-IAP2 suppression to sex-linked Sch-PH pathology, highlighting them as promising therapeutic targets for this life-threatening illness.

## Significance

Over 200 million people are infected with the intravascular parasite *Schistosoma mansoni*, and an estimated 1 to 10 million develop pulmonary arterial hypertension (Sch-PAH), the leading cause of PAH worldwide. Moreover, CDC reports indicate >400,000 cases of infection among US residents, including individuals in the Gulf Coast, Peace Corps, and the military. Beyond its global significance, Sch-PAH shares striking similarities with idiopathic PAH. Since PAH remains incurable, identifying novel molecular targets is critical. This study identifies potential therapeutic avenues, revealing that suppression of endothelial c-IAP2 and upregulation of P2X7R drive chronic pulmonary vascular remodeling. It also provides key insights into how infectious and commensal microbes may imprint a molecular “memory” into the lung endothelium, contributing to long-term vascular dysfunction.

Pulmonary arterial hypertension (PAH) is a life-threatening disease with no cure, characterized by injury and hyperproliferation of lung vascular cells, including endothelial cells (ECs) (1). The hyperproliferation of “abnormal” vascular cells promotes remodeling of pulmonary arteries and arterioles, eventually leading to inflammatory lesions that collectively drive the pulmonary pressure to life-threatening levels (2, 3). Although the primary trigger of noninfectious PAH appears to be multifactorial, several studies indicate that it results from a chronic inflammatory process, such as triggered by infection with the intravascular parasite *Schistosoma mansoni* (4–6). After infecting humans or rodents, *S. mansoni* migrates throughout the cardiovascular system, reaching the mesenteric circulation, where it lays its eggs. Within the mesentery, the eggs either cross the intestinal wall, disturbing the gut microbiome, or migrate to other organs, including the lungs, where they can disrupt lung microbiome homeostasis and cause PAH (7). Affecting >200 million people*, S. mansoni* infection is estimated to result in schistosomiasis-associated PAH (Sch-PAH) in ~1 to 10 million individuals, making this disease the leading cause of PAH globally (8, 9). Sch-PAH also shares key pathological features with other forms of PAH, such as the idiopathic disease, including female prevalence and TGF-β-driven pulmonary vascular remodeling (10, 11). These changes significantly increase pulmonary vascular resistance (PVR), leading to right ventricular (RV) failure and eventual death. Despite its significant global impact and unclear epidemiology in nonendemic countries, schistosomiasis remains in the realm of neglected diseases, with no targeted therapies or specific biomarkers of Sch-PAH early onset (12).

In general, Sch-PAH progression involves a biphasic inflammatory response. During the acute phase of *S. mansoni* infection, a type 1 helper T-cell (Th1) response promotes pathogen killing but also contributes to tissue damage. As the infection persists and thousands of eggs are released, a type 2 helper T-cell (Th2) response dominates, driving tissue fibrosis, excessive vascular cell proliferation, and severe vascular remodeling characteristic of PAH (6, 10). This aligns with a long-standing paradigm in the PAH research field, which suggests that chronic vascular injury leads to a subset of lung ECs overcoming programmed apoptotic cell death, reprogramming, and proliferating, thereby contributing to the development of inflamed vascular lesions (13). Our previous data indicated that during experimental and idiopathic PAH-induced fibrotic vascular remodeling, a subset of lung ECs deficient in expression of the endoprotective proteins Caveolin-1 (Cav-1) and bone morphogenetic protein receptor 2 (BMPR2) survived, proliferated, and contributed to vascular remodeling by stimulating macrophage secretion of profibrotic TGF-β (14). Similarly, our recent findings indicated that exposure to antigenic *S. mansoni* eggs promoted extensive apoptosis of the lung microvasculature and severe inflammatory remodeling, which was also associated with depletion of lung EC-BMPR2 and Cav-1 expression (7). As a scaffold protein and major component of caveolae, Cav-1 modulates several signaling pathways, including death/survival-linked mechanisms, such as the expression of the proinflammatory, damage-associated purinergic receptor, known as the P2X7 receptor (P2X7R) (15).

P2X7R is a ligand-gated ion channel that has been previously implicated in experimental schistosomiasis and a noninfectious model of pulmonary hypertension (PH is used when referring to animal models) (16–18). P2X7R activation occurs in response to mM extracellular adenosine-5-triphosphate (eATP) released after cell stress, injury, and/or death (19). Persistent P2X7R activation induces a massive influx of Ca^2+^ and the formation of a large pore, leading to maturation and secretion of proinflammatory cytokines such as IL-1β and IL-18, and thus, perpetuating inflammation and cell death by inducing pyroptosis and apoptosis (20). Purinergic signaling also affects chronic pulmonary inflammation in response to infectious and *“commensal or innate”* microorganisms, such as those shaping the gut and lung microbiome (21–23). Indeed, gut-lung microbiome dysbiosis, along with cellular damage, has been implicated in the release of eATP, which supports the immune response while also favoring microbial colonization (24). eATP has also been described as regulating gut microbiota through its iron-chelating ability and biofilm dispersion (21). In line with these observations, our recently published data demonstrated that the preclinical Sch-PH model displayed gut and lung microbiome dysbiosis, leading to significant lung microvascular EC apoptosis via an unclear mechanism (4, 7).

Apoptosis is a complex phenomenon that is endogenously inhibited by members of the Inhibitors of Apoptosis Protein (IAP) family (25). The IAP proteins, also known as Baculovirus IAP repeat-containing proteins (BIRC), form a gene family that regulates apoptosis through cell cycle progression and the activation/inhibition of caspases (26). To date, the IAP family comprises eight proteins: XIAP, c-IAP1, c-IAP2, NAIP, Livin, Cp-IAP, Op-IAP, and Survivin (27, 28). All IAPs contain a RING (Really Interesting New Gene) domain, which allows them to function as E3 ubiquitin ligases (26). c-IAP1 and c-IAP2 also contain a unique caspase-recruitment domain (CARD), which facilitates interactions such as caspase-8-mediated apoptosis in tumor necrosis factor (TNF)-mediated signaling (26, 29). c-IAP1 and c-IAP2 also play crucial roles in regulating canonical NF-κB signaling by inhibiting the noncanonical pathway (30), but unlike c-IAP1, c-IAP2 expression is inducible. Since there are no specific therapeutic targets for Sch-PH and the mechanisms underlying the disease are poorly understood, our data shed light on a molecular contribution of c-IAP2 to the pathogenesis of the disease, given its unique autoubiquitination and CARD properties. Therefore, we tested the hypothesis that *S. mansoni* Egg-induced lung microbiome dysbiosis may contribute to increased eATP in lung tissue, leading to overactivation of P2X7R-mediated cell death and suppression of inducible antiapoptotic c-IAP2, thereby sustaining prolonged lung vascular injury and, in turn, driving the inflammatory vascular remodeling that underlies Sch-PH.

## 1. Materials and Methods

Extended methods are included as *SI Appendix*, *Supplementary Material*.

### 1.1. Preclinical Animal Model of Sch-PH.

Sch-PH model was performed as previously established (5, 10, 31). Briefly, 3-mo-old male and female animals received intraperitoneal (IP) sensitization with 240 *S. mansoni* eggs per gram of body weight (bw), followed by an intravenous (IV) tail injection (2.5% isoflurane-anesthetized mice) with 175 eggs/gram/bw 2 wk later (5, 32). Mice were monitored daily, and after 7 d (Day 21; IP/IV Eggs), randomized by sex for subsequent experiments, as approved by the Institutional Animal Care and Use Committee.

### 1.2. *S. mansoni* Cycle and Egg Collection.

All animal studies at Rush University Medical Center were approved by the Institutional Animal Care and Use Committee of the Rush University Medical Center (Department of Health and Human Services animal welfare assurance number A-3120-01; protocol ID: 24-057). *Biomphalaria glabrata*, strain Naval Medical Research Institute, infected with *S*. *mansoni*, was provided by the National institute of Allergy and Infectious Diseases (NIAID) Schistosomiasis Resource Center for distribution through BEI Resources (contract HHSN272201000005I) (33, 34). *S. mansoni* eggs isolated from the livers of infected Swiss-Webster mice (33) were counted, resuspended in sterile Phosphate buffered saline (PBS), and transported in a sealed container.

### 1.3. Novel Conditional Endothelial-Specific BIRC3 Knockout Strain.

Novel animal model of systemic c-IAP1 and conditional endothelial c-IAP2 deletion using *Cdh5* as an EC promoter. Specifically, male and female 8 to 12 wk old heterozygous (HET) and homozygous (HOMO) animals (*Cdh5creER^t2^; c-IAP1^+/^^−^:c-IAP2^+/fl^* and *Cdh5cre-ER^T2^;cIAP1^−/−^,cIAP2^fl/fl^*, respectively) were IP injected with corn oil (vehicle) or 1 mg/d tamoxifen for five consecutive days to induce cre-mediated recombination and c-IAP2 deletion (after 14 d of the last injection) (35).

### 1.4. Genotyping using Polymerase Chain Reaction.

Polymerase chain reaction (PCR) was performed using the REDExtract-N-AMP^TM^ Tissue PCR kit protocol (Sigma, Cat #XNAT-100Rxn). For identification of *Cdhr5*, forward primer (5’-GAT CGC TGC CAG GAT ATA CG-3’) and reverse primer (5’-AAT CGC CAT CTT CCA GCA G-3’) were used. The primers used for *Birc3* were 5’-GTG GTT TCC AAC GGC TTT G-3’; 5’-AAG TCT AGT CAC AGA GGC TCC AGT-3’, and 5’-GAT GGT GGC ACA TGC CTT TAA TCC-3’. PCR products were run on a 2 to 3% agarose gel, and the gels were scanned using a Li-Cor Odyssey CLx (Lincoln, NE).

### 1.5. Assessment of Right Ventricle Systolic Pressure (RVSP) and Hypertrophy (RVH).

A Millar Mikro-Tip catheter transducer (model PVR-1030) was inserted into the RV via the jugular vein of ketamine/Xylazine anesthetized animals (K/X; 100/10 mg/kg body weight; IP). Then, RVSP was calculated using an MPVS-300 system connected to a Powerlab A/D converter (ADInstruments, Colorado Springs). After recordings, blood was collected using 3.8% sodium citrate-treated syringes. The lung lobes were either snap-frozen in liquid nitrogen or inflated with 4% paraformaldehyde (PFA) for histology. Hearts were dissected for RVH analysis (7, 11).

### 1.6. Experimental Rodent Echocardiography.

At day 0 (D0 - baseline) or day 21 (D21) after IV/IP PBS or Egg exposure, heterozygous and homozygous c-IAP2 mice underwent transthoracic echocardiography using Vevo F2 (VisualSonics Inc., Toronto, ON) and a UHF57x transducer. RV free wall thickness (RVFWTH), pulmonary acceleration time (PAT), pulmonary ejection time (PET), heart rate (HR), and tricuspid annular plane systolic excursion (TAPSE) were measured (*SI Appendix*, *Supplementary Material*).

### 1.7. Histological Analysis of Pulmonary Vascular Remodeling and Immunohistochemistry (IHC).

PFA-fixed, paraffin-embedded 5 μm lung sections were used to evaluate CD31, c-IAP2, and P2X7R expression. Fluorescent images were collected using an LSM880 Confocal (Carl Zeiss MicroImaging, Inc). In addition, *Masson’s Trichrome-*stained sections were scanned using an Aperio brightfield automated microscope slide scanner (40×; Leica Aperio AT2), and 10 to 20 microvessels/animal (i.e., vessels with a diameter <100 μm), analyzed using the ImageScope software 12.4.6 (Leica Biosystems) to determine vascular area and thickness, as previously reported (3).

### 1.8. In Situ Apoptosis Assay.

In situ apoptosis was performed as previously published (7), using the terminal deoxynucleotide transferase (TdT)-mediated dUTP nick-end labeling (TUNEL) detection kit (ab206386; Abcam; MA) according to the manufacturer’s instructions. The ratio of TUNEL-positive to total cells (apoptotic index) was measured within the lung microvasculature (vessels > 100 μm).

### 1.9. Enzyme-Linked Immunosorbent Assay (ELISA; Competitive and Multiplex).

BIRC3 (c-IAP2) ELISA was carried out following the protocol from the ELISA Kit (MyBioSource, Cat #MBS7252940). The optical density was then measured using the Benchmark Scientific MR9600-T SmartReader™. Multiplex ELISA was performed according to the manufacturer’s protocol (R&D Systems, Cat# LXSAMSM). The plate was read on the Luminex MAGPIX system, and the results were analyzed using BioTek QuantStudio software and plotted in GraphPad Prism.

### 1.10. Human Microvascular ECs from Lungs (HMVEC-L) and Pulmonary Artery ECs (HPAEC).

HMVEC-L and HPAEC (4th to 7th passage) were obtained from Lonza (Cat No. CC-2527 and CC-2530, respectively), maintained in endothelial basal medium-2 supplemented with endothelial growth medium SingleQuots™ Supplements or MV SingleQuots plus 10% heat-inactivated fetal bovine serum (37 °C and 5% CO_2_). Then, 90 to 100% confluent cells were treated with 10 to 30 ng/mL TNF-α or 50 to 100 ng/mL IFN-γ for 18 h, and cell lysates were analyzed by western blot. Cell morphology was assessed daily by brightfield contrast microscopy. Alternatively, HMVEC-L were exposed to ATP +/− Sm-p40 and A740003. Cell supernatant was used for EV isolation.

### 1.11. Apoptosis Assay by Flow Cytometry.

HMVEC-L seeded in 6-well plates were treated with 3 mM ATP, 30 ng/mL TNF-α, with or without 50 µM A740003 or 200 nM Staurosporine (STS) for 18 h. The cells were washed with PBS and gently detached using trypsin-EDTA 1×. Then, 5 × 10^6^ cells were incubated with the APC Annexin V Apoptosis Detection kit with PI, according to the manufacturer’s protocol (Cat No. 640932; BioLegend). Data were acquired using Gallius, and the events were quantified using Kaluza 2.2 (Beckman Coulter, United States).

### 1.12. Lung Sample Preparation.

Frozen lung tissue and cultured ECs were fully homogenized using cold radioimmunoprecipitation assay (RIPA) buffer containing 1% protease and 0.1% phosphatase inhibitor cocktail. After 20 min at 4 °C, homogenates were centrifuged at 13,538 × g (20 min at 4 ^°^C), and the supernatant was collected for protein measurement. Standard bicinchoninic acid (BCA) protein assay was used to determine the protein concentration of each lung sample using Benchmark Scientific MR9600-T SmartReader™. For Multiplex ELISA, frozen lung tissue was weighed, and 50 to 60 mg was homogenized in buffer containing 1:100 protease inhibitors.

### 1.13. Western Blot (WB).

After running the samples using a gradient SDS-PAGE gel (8 to 12%), proteins were transferred to nitrocellulose membranes, washed twice (TBS-Tween 1× for 5 min), and blocked using 5% milk or 5% BSA for 1 h at RT. Then, primary antibody incubation was carried out overnight at 4 °C or for 2 to 3 h at 37 °C. After washing (2 × 5 min; 1 × 15 min), membranes were incubated for 1 h with secondary HRP-conjugated antibody, washed, and scanned with an ECL kit using a Li-Cor Odyssey CLx (Lincoln, NE). Data were analyzed and normalized to β-actin or GAPDH loading controls using ImageJ software (https://imagej.nih.gov/ij/).

### 1.14. Liquid Chromatography-Mass Spectrometry (LC–MS) for Targeted Metabolomics.

5 μL of the calibrator and sample were injected into an AB SCIEX 5500 QTRAP coupled with an Agilent 1290 UPLC system (samples eluted by Agilent Poroshell column 120 EC-C18 2.7 μm, 2.1 × 100 mm with a flow rate of 450 μL/min; column compartment at 40 °C). LC elution was 99% mobile phase A (0.1% FA in H_2_O) for 1 min, followed by a linear gradient increase of mobile phase B (0.1% FA in ACN) from 1 to 10% in 1 min, then from 10% B to 65% B in 6 min and from 65% B to 90% B in 0.1 min. The column was washed with 90% B (3 min), then re-equilibrated to the initial condition (99% A; 3 min). The autosampler was maintained at 4 °C. MS data were acquired by MRM scan in negative mode. The ESI spray voltage and source temperature were set to −4.5 kV and 450 °C, respectively. Negatively charged analytes and standards were detected by monitoring their transition to signature product ions.

### 1.15. ATP Determination.

Perfused fresh lung tissue (cold saline) was snap frozen in liquid nitrogen. Then, tissue was homogenized in buffer containing 100 µM Suramin to block ectonucleotidase activity and preserve eATP. ATP was then measured as described in the ATP Determination Kit datasheet (A22066, ThermoFisher).

### 1.16. Whole Metagenomic Analysis.

Fresh lung tissue from control and *S. mansoni* IP/IV Egg-stimulated mice was used for metagenomic analysis as previously described (7). Individual samples were transferred to barcoded tubes containing DNA stabilization buffer and analyzed by automatic whole genome sequencing (WGS; Transnetyx, Cordova, United States). DNA samples were sequenced on both Illumina and ONT sequencing platforms. Raw data were analyzed using One Codex software within its Database (https://www.onecodex.com/, accessed: 09/20/25) (36, 37). FASTQ files used in this study will be made publicly available at Sequence Read Archive (SRA) under the submission SUB16225637.

### 1.17. Extracellular Vesicle Isolation and Counting.

Differential centrifugation and filtration methods were adapted to isolate extracellular vesicles (EVs), as previously described (11). Briefly, 3 mL of cell culture supernatant and 200 μL of mouse plasma were centrifuged 2× for 20 min at 1,500 × g (4 °C). Platelet-free plasma was centrifuged at 16,100 × g (20 min; 4°C). Then, the pellet was diluted in double-filtered PBS to determine size and concentration using Nanoparticle Tracking Analysis (NanoSight; Malvern Analytical). Cell numbers were used for normalization. Plasma Cav-1+ EVs were captured using a Cav-1 ELISA Kit, and c-IAP2 expression was assessed by fluorimetry.

### 1.18. Human EDTA-Plasma Samples, ELISA, Data Preparation, and Analysis.

For the present translational analysis, 13 patients with idiopathic PAH (IPAH), 5 with schistosomiasis-associated PAH (SchPAH), and 12 with hepatosplenic schistosomiasis without PAH (SchHSD) were prospectively and consecutively recruited from patients referred for clinically indicated right heart catheterization at the Pulmonary Hemodynamic Assessment Program of the Federal University of São Paulo (Unifesp; São Paulo, Brazil). EDTA-plasma samples were used for BIRC3 ELISA according to the manufacturer’s specifications (MyBioSource, catalog MBS928343). The study was approved by the Unifesp Institutional Ethics Committee (CEP/Unifesp CAAE 49617621.1.0000.5505).

### 1.19. Statistics.

Data were analyzed using One Codex Cloud Platform and GraphPad Prism v10 (GraphPad, La Jolla, CA). Normally distributed data are presented as the x̄ +/− SEM. The Shapiro-Wilk test assessed normality, and the Brown-Forsythe or F-test assessed equality of variances. Parametric statistical analysis was performed using the unpaired Student *t* test between two groups, or one-way or two-way ANOVA, followed by post hoc analysis (Bonferroni, Dunnett, or Tukey Multiple Comparison tests) between >2 groups. The Mann-Whitney test was used for nonparametric analysis. *P* < 0.05 was considered statistically significant.

## 2. Results

### 2.1. Preclinical Sch-PH Model Displays Increased ATP/P2X7R-Mediated Signaling in Lung Microvascular EC.

**Fig. 1.**
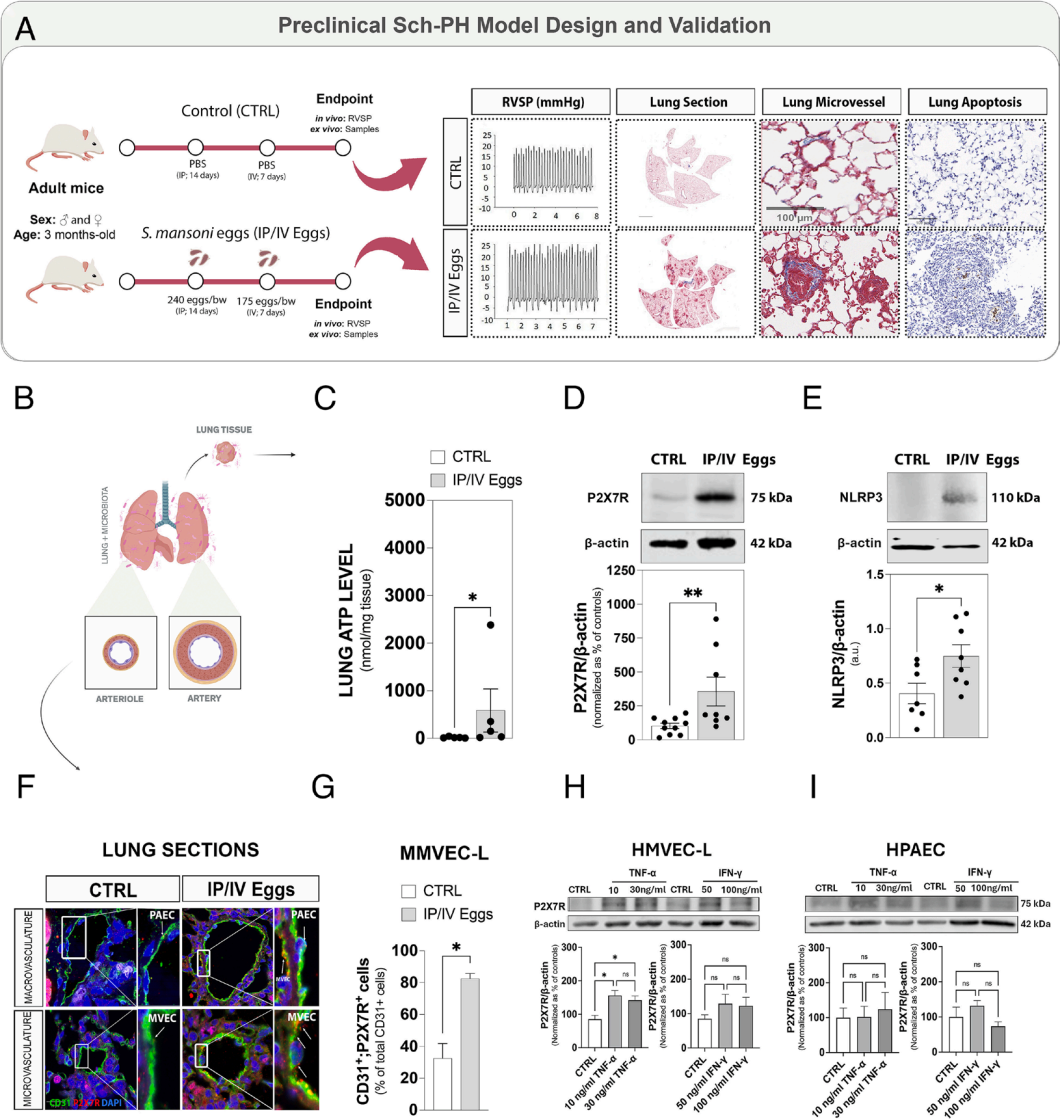
Preclinical schistosomiasis-associated pulmonary hypertension model displays increased ATP/P2X7R-mediated signaling in lung microvascular endothelial cells: Preclinical schistosomiasis-associated pulmonary hypertension (Sch-PH) animal model was induced in 3-mo-old mice by intraperitoneal (IP) sensitization using 240 *S. mansoni* eggs/gram of body weight (bw), followed by intravenous (IV) tail injection with 175 eggs/gram bw after 2 wk (IP/IV Eggs). After 7 d, male and female mice were randomized and used for in vivo hemodynamic analysis (RVSP = Right Ventricular Systolic Pressure in mmHg), for assessment of the RV hypertrophy (RVH) using the Fulton Index [RVH = RV/(left ventricle + septum)], and tissue procurement for histological analysis of pulmonary vascular remodeling and cellular apoptosis in lung tissue sections using *Masson’s Trichrome* and TUNEL as staining, respectively (*A*). Schematic image demonstrating sample collection at end-stage procurement, where whole lung tissue samples were collected from male and female mice and used for immunohistochemistry, ATP quantification, and western blot. The inset shows a graphic representation of a pulmonary microvessel (arteriole) and a macrovessel (artery) (*B*). Lung ATP levels (nmol/mg tissue) measured by luminescence assay using whole lung lysates in control (CTRL; white bars) and IP/IV Egg-exposed animals (light gray bars; *C*). Western blot analysis of P2X7R and NLRP3 expression in whole lung lysates from CTRL and IP/IV Egg-exposed animals (*D* and *E*). Immunohistochemistry analysis of the micro- and macrovasculature of control and IP/IV Egg-exposed animals stained with CD31 (green), DAPI-stained nuclei (blue), and P2X7R (red) and quantification of CD31 and P2X7R positive cells (*F* and *G*; MMVEC-L = murine microvascular endothelial cells - lung). Western blot analysis of P2X7R expression in human lung microvascular endothelial cells (HMVEC-L) and human lung pulmonary arterial endothelial cells (HPAEC-L) treated with TNF-α (10 to 30 ng/mL) and IFN-γ (50 to 100 ng/mL) (*H* and *I*). β-actin was used as a control. Normally distributed data were analyzed using Student’s *t* test or one-way ANOVA, and nonparametric analysis used the Mann–Whitney test (n = 8 to 10 animals/group - at least 4 to 5 mice of each sex; n = 3 different cultures; ns = nonsignificant; *P < 0.05; **P < 0.01).

Our recent findings in the preclinical Sch-PH animal model revealed gut and lung microbiome dysbiosis, along with significant lung microvascular apoptosis, specifically in the intima layer, though the underlying mechanism remained unclear (4, 7). Dysfunctional gut and lung microbiome composition can significantly dampen circulating levels of anti-inflammatory microbial metabolites such as short-chain fatty acids (SCFAs), whereas it can also increase levels of damage-associated molecular patterns, such as ATP, contributing to a sustained inflammatory response. To evaluate the contribution of circulating SCFAs in Sch-PH, we compared plasma propionate, butyrate, isovalerate, and acetate from validated preclinical IP/IV Egg-exposed with vehicle control animals, with MassSpec analysis revealing no significant differences between groups (*SI Appendix*, Fig. S1*A*). On the contrary, lung ATP level was significantly increased in IP/IV Egg-exposed animals (Fig. 1 *A–C*). Functional pathway analysis of gut-derived metadata (7) using MetaCYC indicated no significant differences between groups, whereas lung-derived metadata pointed to reduced aerobic respiration (cytochrome C) in IP/IV Egg-exposed animals (*SI Appendix*, Fig. S1 *B* and *C*), consistent with changes in lung eATP level. The IP/IV Egg animal model also exhibited a marked increase in lung microvascular injury (7) (Fig. 1*A*), a well-established source of eATP. In line with these findings, western blot analysis of lung tissue revealed significant upregulation of both purinergic P2X7R and NLRP3, key components of the canonical ATP/P2X7R signaling pathway (Fig. 1 *D* and *E*). Notably, this increase was more pronounced in the microvasculature than in larger arterial vessels (Fig. 1 *F* and *G*). Further in vitro experiments using TNF-α and IFN-γ confirmed the selective expression of P2X7R in the pulmonary microcirculation induced by inflammation. Specifically, TNF-α, but not IFN-γ, induced P2X7R expression in HMVEC-L compared to HPAEC (Fig. 1 *H* and *I*). Overall, the data indicated that *S. mansoni* Egg-associated inflammation increases eATP and preferentially induces endothelial P2X7R expression in the pulmonary microvasculature during Sch-PH, where high levels of apoptosis were also observed (7).

### 2.2. Increased Lung P2X7R and Reduced c-IAP2 Contribute to Lung Microvascular EC Apoptosis in Sch-PH.

**Fig. 2.**
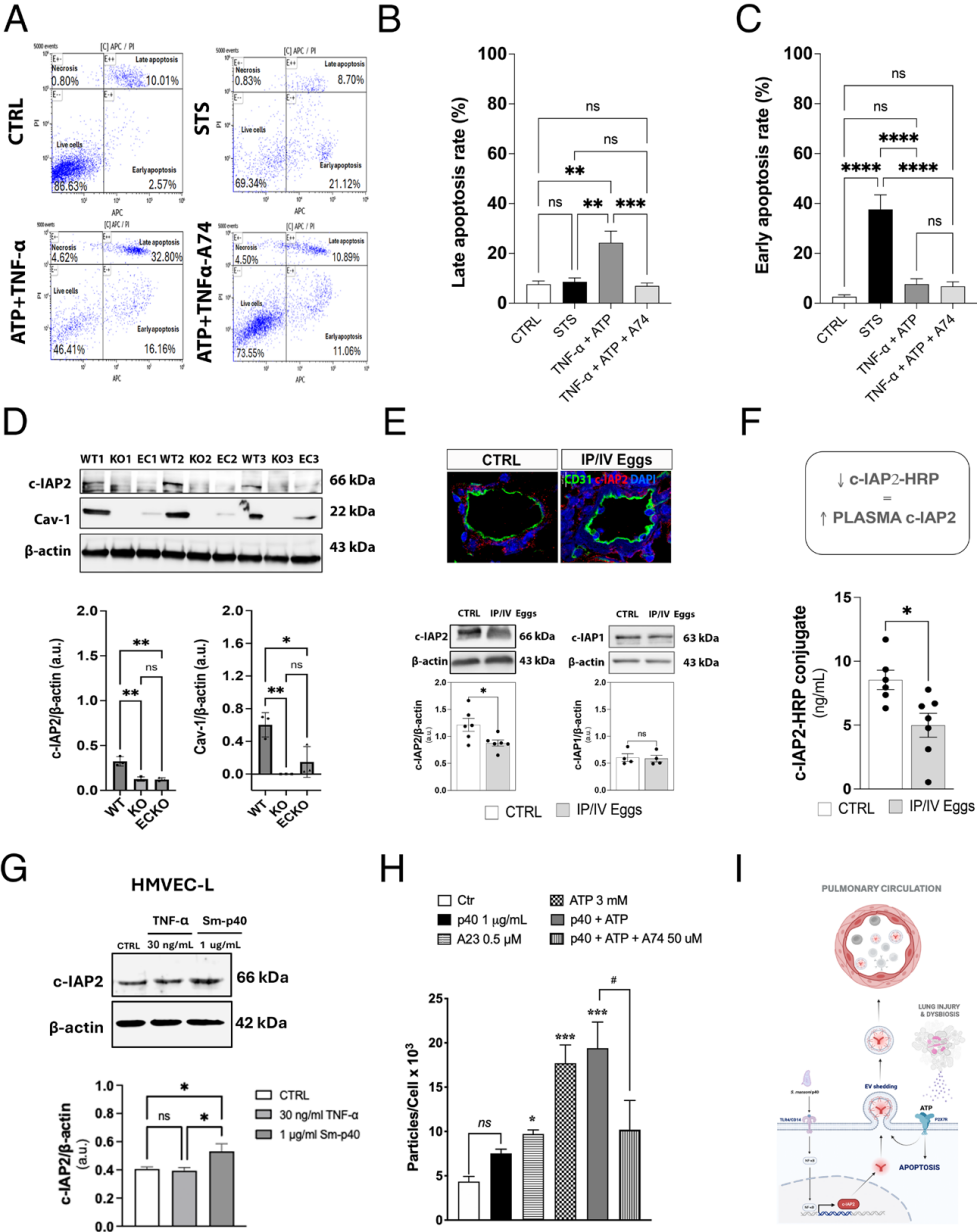
Increased lung P2X7R and reduced c-IAP2 contribute to lung microvascular EC apoptosis in schistosomiasis-associated pulmonary hypertension: Flow cytometry analysis of P2X7R-mediated late and early apoptosis in human lung microvascular endothelial cells (HMVEC-L) after 18 h of treatment with vehicle control (CTRL) or 30 ng/mL TNF-α + 3 mM ATP with or without 50 μM P2X7R antagonist A740003 (A74). Staurosporine (STS; 200 nM) was used as a positive control (*A*–*C*). Western Blot analysis of c-IAP2 and Caveolin-1 (Cav-1) expression in the whole lung lysates of wildtype (WT), global Cav-1 KO (KO), and EC-specific Cav-1KO (ECKO) mice (*D*). Immunohistochemistry of c-IAP2 expression (red), CD31 (green), and DAPI-stained nuclei (blue) in CTRL and IP/IV *S. mansoni* egg-exposed mice (IP/IV Eggs) and western blotting of c-IAP2 and c-IAP1 expression in whole lung lysates of CTRL and IP/IV Eggs mice (*E*). Competitive c-IAP2 enzyme-linked immunosorbent assay (ELISA) in the plasma samples of CTRL and IP/IV Eggs mice (*F*). Western Blot analysis of c-IAP2 expression in HMVEC-L in CTRL conditions or exposed to 10 ng/mL TNF-α + 5 mM ATP, or 1 ug/mL *S. mansoni* egg protein 40 (Sm-p40) + 30 ng/mL TNF-α + 5 mM ATP (*G*). β-actin was used as a control. Untreated HMVEC-L or A23187-, ATP-, A740003-, and Sm-p40-treated cells were also used to quantify extracellular vesicles (*H*). Schematic figure demonstrating *that S. mansoni* egg exposure may activate the NFκB inflammatory cascade via the TLR4/CD14-mediated signaling pathway, simultaneously with exogenous activation of P2X7R by extracellular ATP (eATP), leading to the depletion and subsequent release of c-IAP2 into the pulmonary circulation (*I*). Normally distributed data were analyzed using Student’s *t* test or one-way ANOVA, and nonparametric analysis used the Mann–Whitney test (n = 6 to 7 animals/group - at least 3 of each sex, n = 3 different cultures; ns = nonsignificant; *P < 0.05; **P < 0.01; ***P <0.001; ****P < 0.0001).

ATP-induced P2X7R activation leads to massive influx of Ca^2+^, EV shedding, cytokine secretion, and cell death (20). In vitro, P2X7R inhibition in HMVEC-L was sufficient to prevent TNF-α/eATP-mediated late apoptosis (Fig. 2 *A–C*). P2X7R has been shown to colocalize with Cav-1 in healthy lung vasculature (38–40). Reduced lung EC-Cav-1 has been extensively demonstrated to contribute to lung endothelial dysfunction and apoptosis in PAH, including by regulating survivin; (41) however, its impact on other IAP members remained largely unclear. An initial analysis of IAP genes in isolated lung ECs from *Cav1*^−/−^ mice found upregulation of c-IAP2 (BIRC3) and survivin (BIRC5) expression compared to WT cells (*SI Appendix*, Fig. S2*A*). In contrast to c-IAP2, survivin lacks the RING and CARDs, which regulate protein autoubiquitination, turnover, and their ubiquitin ligase activity, making inducible c-IAP2 unique (27, 28). Despite increased mRNA levels, the lung tissue of global Cav-1 knockout (KO) and EC-specific Cav-1 knockout (ECKO) mice were deficient in c-IAP2 expression (Fig. 2*D*), similar to what is observed in the lungs from Sch-PH mice (Fig. 2*E*). Structurally similar to c-IAP2, the expression of constitutive c-IAP1 remained unchanged between groups (Fig. 2*E*). Reduced lung endothelial c-IAP2 expression was also confirmed by IHC (Fig. 2*E*). Measurement of HRP-conjugated c-IAP2 level using a competitive ELISA indicated its reduction in plasma samples from Sch-PH mice compared to controls (Fig. 2*F*). As an inversely proportional assay, it revealed a higher c-IAP2 level in the plasma of Sch-PH mice. To further investigate the molecular mechanism underlying c-IAP2 depletion, HMVEC-Ls were exposed to TNF-α, ATP, and the *S. mansoni* major antigen p40 (Sm*-*p40). Although no significant effect on c-IAP2 expression was observed in HMVEC-L after 18 h of TNF-α + ATP treatment alone, acute Sm*-*p40 exposure significantly increased c-IAP2 expression (Fig. 2*G*). Previously, we observed that injured lung ECs shed Cav-1 protein into the plasma of the Hypoxia-Sugen animal model of PH as a component of EVs. Recently, we also observed an increase in circulating EVs in IP/IV Egg-exposed animals. Since c-IAP2 expression has been reported in EVs from cancer cell lines (42), we next evaluated whether Sm*-*p40 and P2X7R would play a role in endothelial-derived EV shedding. As previously reported, acute Sm*-*p40 exposure alone did not induce EV shedding from HMVEC-L. However, eATP alone or in combination with Sm-p40 induced a higher level of EV shedding than controls; an effect prevented by P2X7R inhibition (Fig. 2 *H* and *I*). Therefore, these data indicate that lung EC-c-IAP2 expression is potentially protective against the development of Sch-PH, and its depletion is, in part, due to its release into the plasma as a response to *S. mansoni* Egg exposure.

### 2.3. Novel EC-c-IAP2^−/−^ Model Displays Increased Lung Microvascular P2X7R Expression and Spontaneous PH-Like Features.

**Fig. 3.**
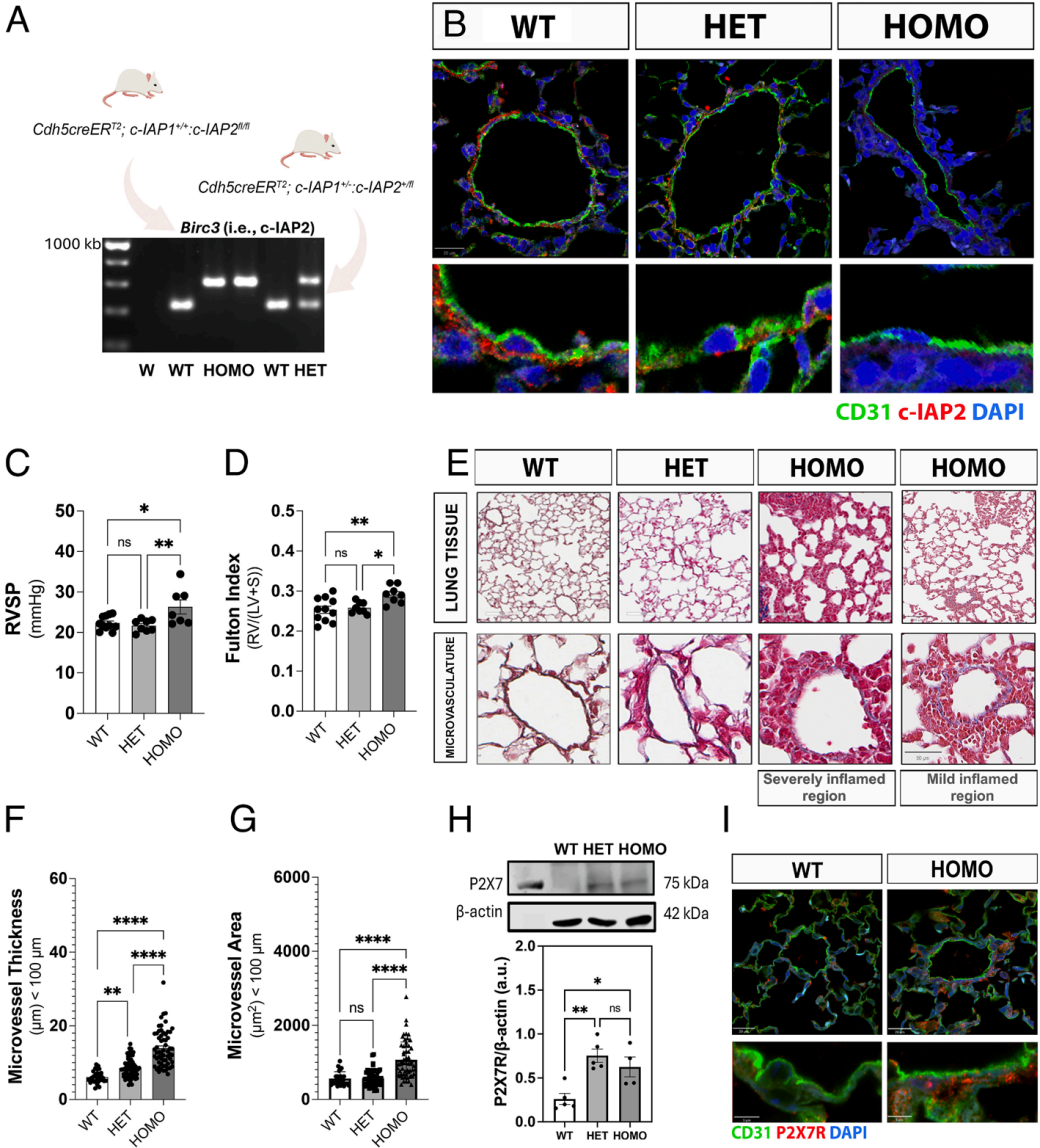
Novel endothelial-specific c-IAP2 knockout mice model displays spontaneous pulmonary hypertension-like features and increased lung microvascular endothelial P2X7R expression: Polymerase chain reaction (PCR) was used to validate the presence or absence of floxed *Birc3* (c-IAP2) in wild-type control (WT), heterozygous (HET), and homozygous (HOMO) mice (*Cdh5creER^t2^;c-IAP1^+/^^−^;c-IAP2^+/fl^* and *Cdh5cre-ER^t2^;cIAP1^−/−^,cIAP2^fl/fl^,* respectively). W = water control (*A*). Immunohistochemistry analysis of CD31 (green), DAPI-stained nuclei (blue), and c-IAP2 expression (red) in lung sections of WT, HET, and HOMO mice (*B*). Right Ventricular Systolic Pressure (RVSP; mmHg) and Fulton Index [Right Ventricular Hypertrophy (RVH) measured by right ventricle/(left ventricle + septum)] were measured in WT (white bars), HET (light gray bars), and HOMO (dark gray bars) mice (*C* and *D*). *Masson’s Trichrome* staining of lung sections from WT, HET, and HOMO mice showing microvessels and lung parenchyma (*Top* panels; Scale bar, 100 μm), with higher-magnification images of the microvasculature in healthy, mildly inflamed, or severely inflamed regions (*Bottom* panels; Scale bar, 50 μm) (*E*). Microvessel thickness (μm; *F*) and area (µm^2^; *G*) were quantified from vessels smaller than 100 μm diameter in the lung sections of WT, HET, and HOMO mice. Western blot analysis of P2X7R expression in whole lung lysates of WT, HET, and HOMO mice (*H*). Immunohistochemistry of P2X7R expression (red), CD31 (green), and DAPI-stained nuclei (blue) in WT and HOMO mice (*I*). Normally distributed data were analyzed using the Student’s *t* test or one-way ANOVA (n = 4 to 7 animals/group - at least 3 mice of each sex; ns = nonsignificant; *P < 0.05; **P < 0.01; ***P < 0.001; ****P < 0.0001).

To further investigate the causal relationship of EC-c-IAP2 expression to pulmonary vascular disease and its contribution to vascular homeostasis, we generated a novel endothelial-specific *c-IAP1^−/−^:c-IAP2^fl/fl^* mouse strain under transcriptional control by *Cdh5creER^t2^*; validated through PCR, IHC, and western blot (Fig. 3 *A* and *B* and *SI Appendix*, Fig. S2*B*). Hemodynamic analysis revealed a significant increase in RVSP (Fig. 3*C*) and RVH (Fig. 3*D*) in HOMO compared to HETs and WT controls. Additionally, EC-c-IAP2 deletion induced spontaneous inflammatory pulmonary vascular remodeling (Fig. 3 *E–G*) and increased lung microvascular EC-P2X7R expression (Fig. 3 *H* and *I*). No significant difference in lung total or phosphorylated-Cav-1 expression was observed among groups (*SI Appendix*, Fig. S2 *C* and *D*). Together, these data suggest that genetic ablation of EC-c-IAP2 expression increases P2X7R and contributes to the development of a mild but spontaneous PH-like phenotype, indicating a putative connection between lung P2X7R and c-IAP2 signaling pathways.

### 2.4. *S.* *Mansoni* Egg Exposure Exacerbates PH in Novel Endothelial-Specific c-IAP2 Ablated Mice.

**Fig. 4.**
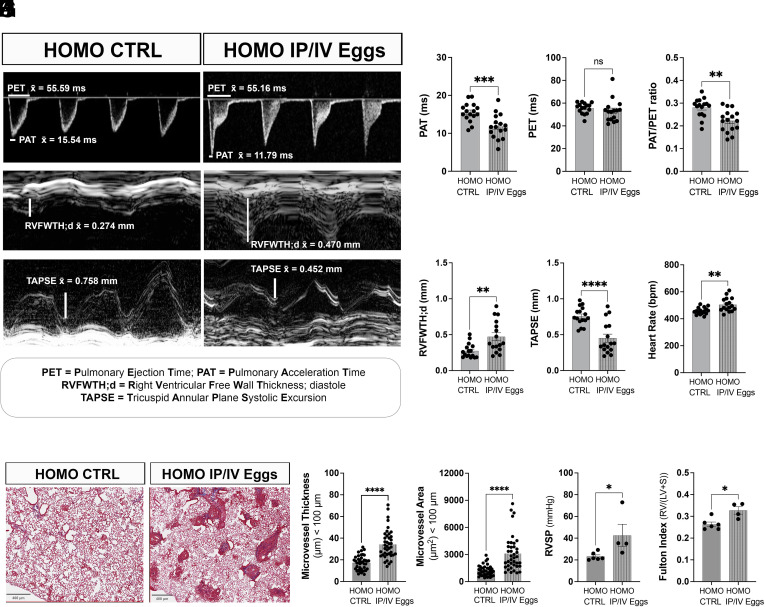
*S. mansoni* Egg exposure exacerbates pulmonary hypertension in novel endothelial-specific c-IAP2 ablated mice: (*A*) Representative echocardiography images displaying right ventricle (RV) function and structural differences between homozygous (HOMO; *Cdh5cre-ER^t2^;cIAP1^−/−^,cIAP2^fl/fl^*) control mice (CTRL) and exposed to *S. mansoni* eggs (HOMO IP/IV Eggs). Bars indicate the average of each parameter measured. Specifically, the *Top* panel displays pulmonary acceleration time (PAT) and ejection time (PET) in milliseconds (ms; graphically analyzed in *B*,*C*, and *D*); the *Middle* panel displays the RV free wall thickness (RVFWTH) in millimeters (mm), measured at end-diastole (graphically analyzed in *E*); and the *Bottom* panel shows the tricuspid annular plane systolic excursion (TAPSE; mm; graphically analyzed in *F*). Heart rates [beats per minute (bpm)] between groups are shown in *G* (n = 16 animals/group; 50% of each sex). *Masson’s Trichrome* staining of lung tissue sections (Scale bar, 400 μm; *H*) was used to determine microvessel thickness (µm; *I*) and area (µm^2^; *J*) in vessels smaller than 100 μm diameter. Right Ventricular Systolic Pressure (RVSP; *K*) and Fulton Index (Right Ventricular Hypertrophy (RVH; *L*); right ventricle/(left ventricle + septum) measurements were also performed. Data were analyzed using Student’s *t* test (n = 4 to 6 animals; ns = nonsignificant; *P < 0.05; **P < 0.01, ***P < 0.001, ****P < 0.001).

To address the relevance of EC-c-IAP2 expression for the RV function and hypertrophy in Sch-PH, *Cdh5creER^t2^;c-IAP1^−/−^:c-IAP2^fl/fl^* underwent echocardiography before (baseline; D0) and 21 d (D21) after PBS or IP/IV Egg exposure (Fig. 4*A*). RVSP and RVH were also assessed. 21D egg stimulus reduced PAT and PAT/PET ratio compared with controls (Fig. 4 *B–D*), indicating increased PVR. No differences in PET alone were observed (Fig. 4*C*). Egg stimulus also reduced TAPSE and increased RVFWTH (Fig. 4 *E* and *F*), HR (Fig. 4*G*), pulmonary microvascular area and thickness (Fig. 4 *H–J*), RVSP and RVH (Fig. 4 *K* and *L*), confirming exacerbated PH compared to PBS controls. Together, the data suggest an essential role of EC-c-IAP2 expression in the severity of Sch-PH.

### 2.5. Pharmacological Inhibition of P2X7R Ameliorates Pulmonary Vascular Remodeling and Prevents RVSP Increase in Sch-PH Animals but does not Prevent RVH or Fully Restore C-IAP2 Expression.

**Fig. 5.**
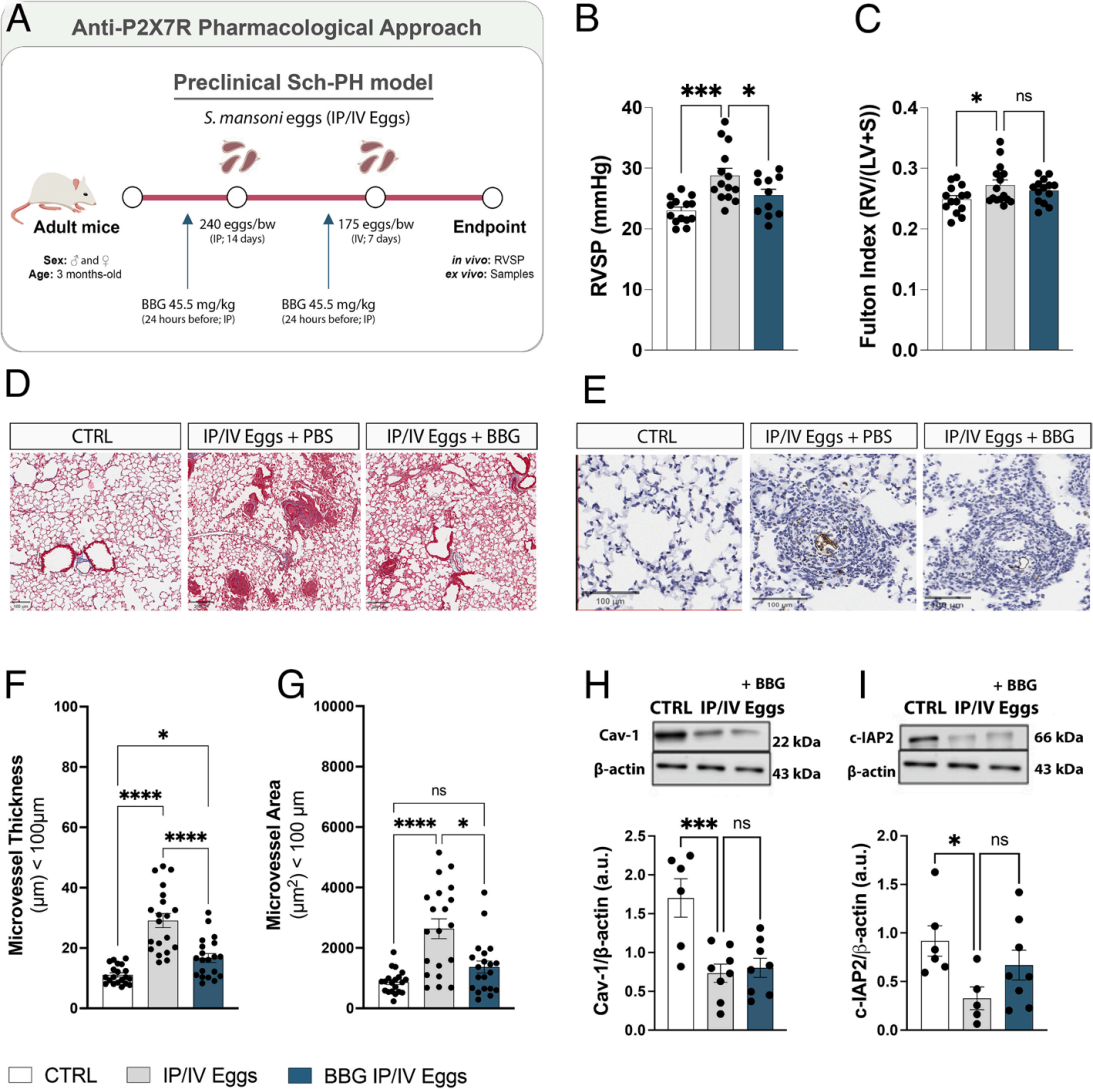
Pharmacological inhibition of P2X7R ameliorates pulmonary vascular remodeling and prevents RVSP increase in Sch-PH animals but does not prevent RVH or fully restore c-IAP2 expression: Figure representation of the pharmacological approach using two intraperitoneal doses of P2X7R inhibitor brilliant blue G (BBG; 45.5 mg/kg) in the preclinical schistosomiasis-associated pulmonary hypertension (Sch-PH) animal model. Sch-PH was induced in 3-mo-old mice by intraperitoneal (IP) sensitization using 240 *S. mansoni* eggs/gram of body weight (bw), followed by intravenous (IV) tail injection with 175 eggs/gram bw after 2 wk (IP/IV Eggs) (*A*). Right Ventricular Systolic Pressure (RVSP) and Fulton Index [Right Ventricular Hypertrophy (RVH); right ventricle/(left ventricle + septum)] in control (CTRL; white bars), IP/IV Eggs (light gray bars), and BBG IP/IV Eggs mice (blue bars) (*B* and *C*). Representative micrographs displaying *Masson’s Trichrome* and TUNEL staining in lung sections of CTRL, IP/IV Eggs, and BBG IP/IV Eggs mice (*D* and *E*). Micrographs from 2 to 3 animals per group were used to quantify microvessel thickness (μm; *F*) and area (μm^2^; *G*) from vessels smaller than 100 μm diameter in the lung sections. Western blot analysis in whole lung tissue lysates of CTRL, IP/IV Eggs, and BBG IP/IV Eggs mice was used to quantify lung Caveolin-1 (Cav-1) (*H*) and c-IAP2 (*I*) expression. β-actin was used as a control. Normally distributed data were analyzed using Student’s *t* test or one-way ANOVA (n = 8 to 10 animals/group - at least 4 to 5 mice of each sex; ns = nonsignificant; *P < 0.05; **P < 0.01; ***P < 0.001; ****P < 0.0001).

To test whether P2X7R would serve as a potential pharmacological target to prevent or attenuate Sch-PH in a preclinical setting, animals were IP injected with two doses of 45.5 mg/kg BBG 24 h before the IP and IV *S. mansoni* Egg injections (Fig. 5*A*). Pharmacological treatment demonstrated efficacy in preventing RVSP increase (Fig. 5*B*) but showed no significant effect in preventing RVH in the Sch-PH animal model (Fig. 5*C*). BBG treatment also reduced pulmonary vascular remodeling and the presence of apoptotic cells within the lung tissue of the *S. mansoni* Egg-exposed group compared with the animals stimulated only with eggs (Fig. 5 *D–G*) but was not able to fully rescue lung Cav-1 or c-IAP2 expression caused by *S. mansoni* Egg-exposed mice (Fig. 5 *H* and *I*). Overall, the data revealed P2X7R as a potential pharmacological target for preventing Sch-PH development.

### 2.6. Sex-Linked Variations in the Preclinical Sch-PH Model May Influence the Efficacy of Anti-P2X7R Pharmacological Approach.

**Fig. 6.**
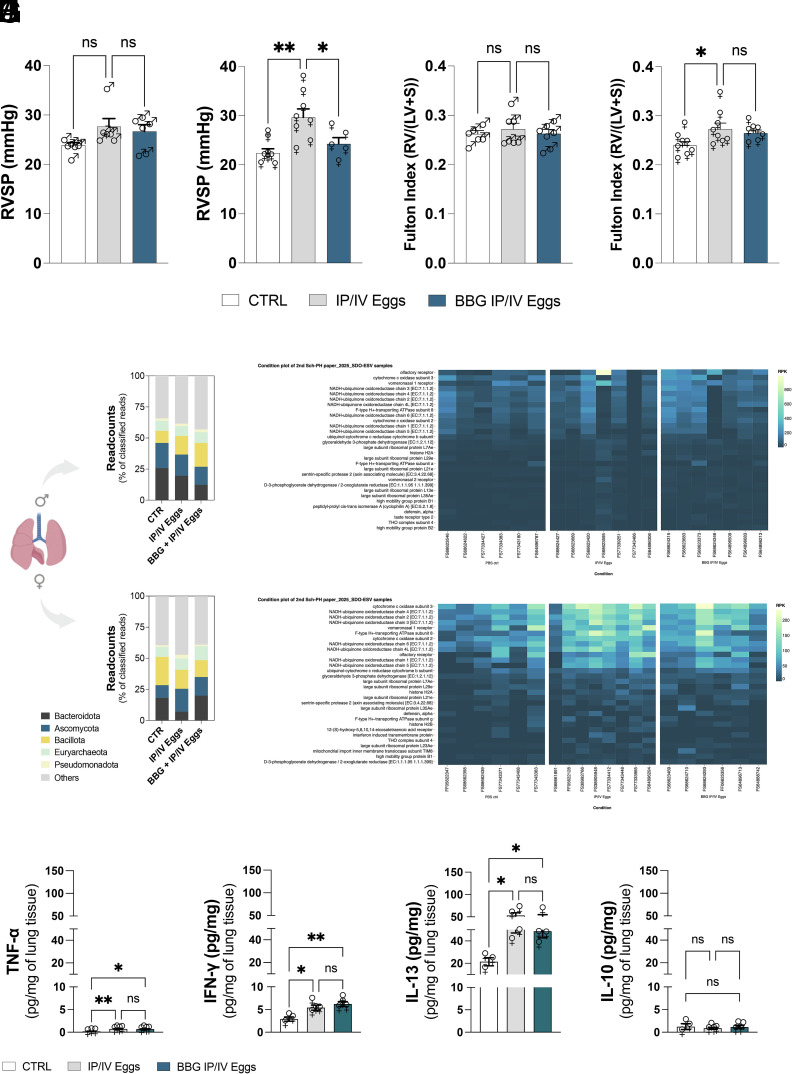
Sex-linked variations in the novel EC-c-IAP2 ablated and preclinical schistosomiasis-associated pulmonary hypertension mice may influence the efficacy of the pharmacological approach targeting P2X7R. Right Ventricular Systolic Pressure (RVSP) and Fulton Index [Right Ventricular Hypertrophy (RVH) = RV/(left ventricle + septum)] in control (CTRL; white bars), exposed to IP/IV Eggs (schistosomiasis-associated pulmonary hypertension; Sch-PH model; light gray bars), and +/− two doses of the P2X7R inhibitor Brilliant Blue G (BBG; 45.5 mg/kg; blue bars) of both male (**♂**) and female (♀) (*A*–*D*) mice. Schematic image of collected lung tissue being analyzed for metagenomics and associated metabolic pathways in male and female CTRL, IP/IV Eggs, and BBG IP/IV Eggs. Readcounts showing the % of classified reads of the Phylum Bacteroidota (dark blue), Ascomycota (light blue), Bacillota (dark yellow), Euryarchaeota (light green), Pseudomonadota (light yellow), and Others (light gray). KEGG Orthology RPK (Reads Per Kilobase) data from a metagenomic analysis, comparing gene expression across three distinct biological conditions and between sexes (*E* and *F*). TNF-α, IFN-γ, IL-13, and IL-10 (pg/mg of lung tissue) in whole lung lysates from CTRL mice or exposed to *S. mansoni* IP/IV Eggs +/− BBG (45.5 mg/kg; *G*–*J*). White bars = CTRL, gray bars = IP/IV Eggs, blue bars = BBG IP/IV Eggs mice. Data were analyzed by one-way ANOVA or Student’s *t* test (n = 4 to 8 animals/group - at least 3 mice of each sex; ns = nonsignificant; *P <0.05; **P < 0.01, ***P < 0.001, ****P < 0.001).

Our data indicate that only female animals displayed significant RVSP and RVH in the preclinical Sch-PH model and that RVH was not prevented by BBG (Fig. 6 *A–D*). Sex-related differences can influence the microbiome and metabolites, including purine derivatives. Accordingly, lung microbiome composition followed different patterns between sexes, with the overall percentage of the Phylum Bacillota tending to a bigger abundance in females and reduced after Egg exposure; an effect not rescued by BBG (Fig. 6*E* and *SI Appendix*, Fig. S3). KEGG Orthology data from a metagenomic analysis indicated a distinct transcriptional landscape that suggests sex-specific responses (Fig. 6*F*). While some genes exhibited moderate expression, many reverted to lower activity. On the other hand, female IP/IV (center block) exhibits consistently higher transcriptional activity across a broad array of genes compared to PBS controls and BBG, particularly for genes associated with respiration (e.g., succinate dehydrogenase, ATPase subunits) and protein synthesis (ribosomal proteins L10e and L3e). This pattern suggests a metabolically active microbial community, potentially thriving under favorable host-associated conditions; a profile not observed in the males. Moreover, in the female BBG-treated group, the overall expression was relatively low, suggesting a community with limited metabolic engagement. A few genes, such as cytochrome c oxidase subunit 3 and NADH-ubiquinone oxidoreductase chain 1, showed slightly elevated expression; however, the general trend indicates a low-energy state. To further evaluate the contribution of inflammation to BBG treatment in females, a multiplex ELISA was performed. After IP/IV Egg exposure, females’ lungs showed elevated TNF-α, IFN-γ, and IL-13 levels compared with PBS controls, but BBG treatment did not alter these levels (Fig. 6 *G–I*). The simultaneous increase in Th1-associated TNF-α and IFN-γ, along with Th2-associated IL-13, suggests a mixed or transitional inflammatory phase. An unchanged IL-10 level indicates the disease did not fully progress to a chronic regulatory phase (Fig. 6*J*). Overall, these data suggest that the observed protective effects of the BBG pharmacological approach tested are unlikely to be mediated by modulation of these cytokines and instead are more consistent with direct P2X7R inhibition in the pulmonary microcirculation.

### 2.7. Investigating Plasma c-IAP2 as a Potential Biomarker of P2X7R-mediated Pulmonary Vascular Disease in Female Animals and PAH Patients.

**Fig. 7.**
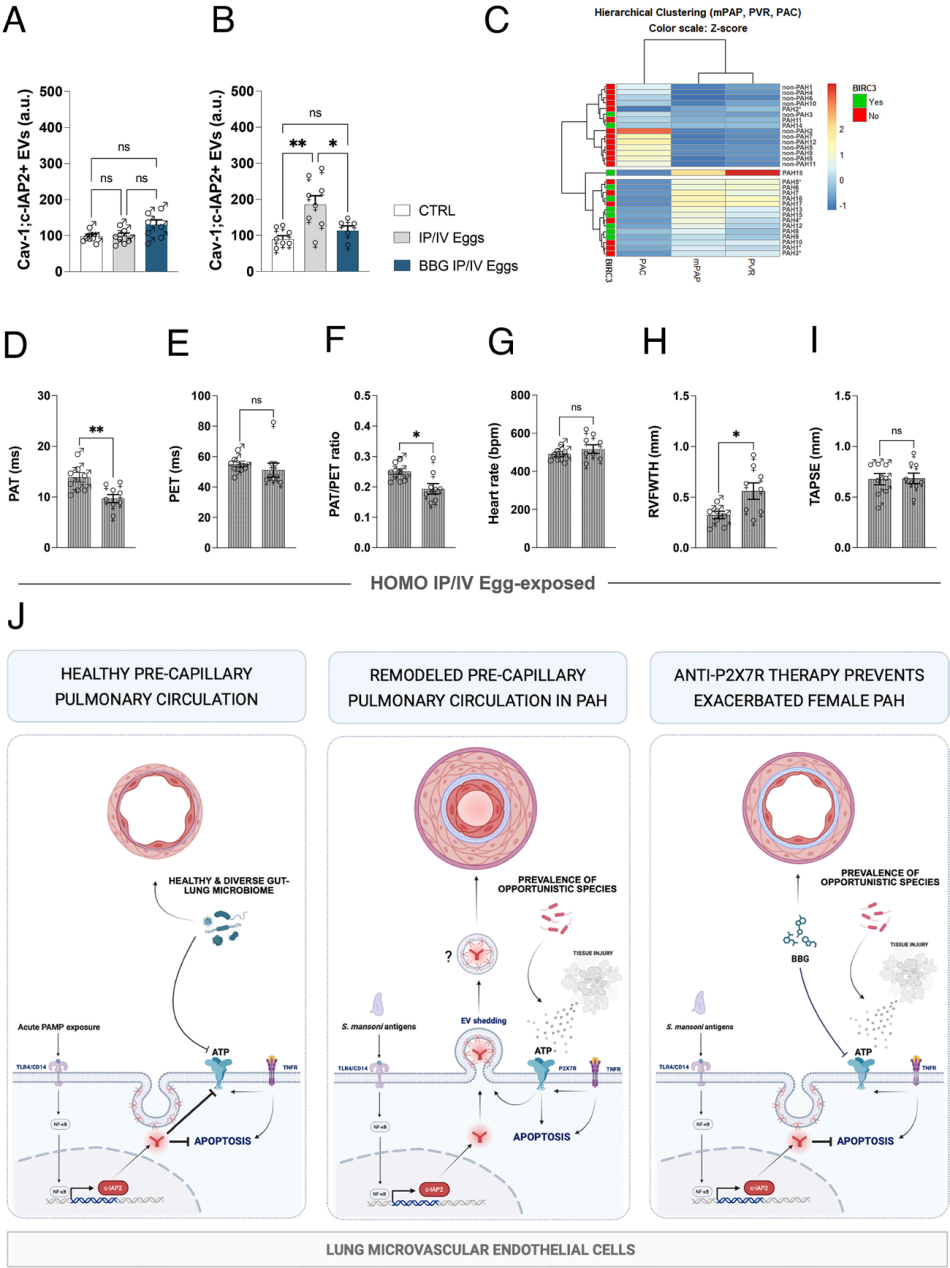
Investigating plasma c-IAP2 as a potential biomarker of P2X7R-mediated pulmonary vascular disease in female animals and PAH patients: Plasma extracellular vesicles positive for c-IAP2 and Caveolin-1 expression (Cav-1; cIAP2+ EVs) in male (**♂**) and female (♀) control (CTRL; white bars), *S. mansoni* IP/IV Eggs (light gray bars, and brilliant Blue G (BBG; blue bars) IP/IV Eggs (2 doses of 45.5 mg/kg; *A* and *B*) mice. Unsupervised hierarchical clustering was performed using standardized (Z-score) values of mean pulmonary arterial pressure (mPAP), pulmonary vascular resistance (PVR), and pulmonary arterial compliance (PAC). Euclidean distance and Ward’s linkage method were applied to generate the dendrogram. Three clusters were defined (k = 3). The heatmap displays Z-score–normalized values (red = above mean; blue = below mean). The side annotation indicates BIRC3 detection status (green = Yes; red = No). Patient labels correspond to unidentified patient IDs and diagnostic groups (non-PAH: 12 SchHSD patients; PAH: 5 SchPAH and 13 IPAH patients). SchPAH indicated by * (*C*). Pulse wave doppler echocardiography was used to measure pulmonary acceleration time (PAT; *D*), pulmonary ejection time (PET; *E*), and the PAT/PET ratio in homozygous (HOMO; *Cdh5cre-ER^t2^;cIAP1^−/−^,cIAP2^fl/fl^*; striped light gray bars) IP/IV egg-exposed mice of both sexes (*F*). Heart rate in HOMO IP/IV egg-exposed mice of both sexes (*G*). Right ventricle free wall thickness (RVFWTH) measured at the end-diastole and tricuspid annular systolic excursion (TAPSE) were calculated and measured in M-mode echocardiograms (*H* and *I*). Data were analyzed by one-way ANOVA or Student’s *t* test (n = 8 animals/group; ns = nonsignificant; *P <0.05; **P < 0.01). Representation of the healthy precapillary pulmonary circulation, remodeled precapillary pulmonary circulation during Sch-PH, and anti-P2X7R therapy. In a healthy state, the pulmonary circulation harbors a diverse lung microbiome that protects the vascular system. When endothelial cells (ECs) are exposed to pathogen-associated molecular patterns (PAMPs) via the TLR4/CD14 signaling pathway, the NFĸB inflammatory cascade. This cascade leads to c-IAP2 expression, which inhibits apoptosis and maintains normal EC function. However, during Sch-PH, disruption of the lung microbiome by *S. mansoni* infection and egg antigens can activate the TLR4/CD14 signaling pathway, leading to c-IAP2 induction. Additionally, microbiome dysbiosis and infection-induced tissue injury can contribute to increased extracellular ATP, which can then activate P2X7R. Instead of c-IAP2 inhibiting P2X7R and apoptosis, it is now released into the circulation via extracellular vesicles, and the progression of the disease continues through overactivation of P2X7R and dysfunction of EC apoptosis. When BBG is used as a treatment, P2X7R is inhibited, preventing activation from tissue injury caused by opportunistic species. However, *S. mansoni* egg antigens can still activate the TLR4/CD14 signaling pathway and the NFĸB inflammatory cascade, inducing c-IAP2 but, overall, ameliorating Sch-PH (*J*).

c-IAP2 is not commonly detected in human plasma of healthy individuals (https://www.proteinatlas.org/human proteome/blood/proteins+detected+in+ms), but it has been found in cell supernatants, lung sputum of asthmatic patients, and cancer-associated EVs (42). Our data showed that c-IAP2 was detected in plasma Cav-1-EVs, previously reported as increased in PAH patients. Specifically, Cav-1; c-IAP2+ EVs were elevated in female IP/IV Egg-exposed animals, which was prevented by BBG (Fig. 7 *A* and *B*). Additionally, plasma c-IAP2 (BIRC3) ELISA performed in a small group of female non-PAH (SchHSD) and PAH (IPAH and SchPAH) patients revealed a similar trend. Specifically, unsupervised hierarchical clustering using standardized hemodynamic parameters (mPAP, PVR, and PAC) revealed two main clusters: one enriched for non-PAH patients, characterized by lower mPAP and PVR with relatively preserved PAC; and another enriched for PAH patients displaying a more severe hemodynamic phenotype (higher mPAP and PVR, reduced PAC). Notably, c-IAP2 detection was predominantly observed in the PAH cluster. Consistent with this pattern, a chi-square test of independence demonstrated a statistically significant association between diagnostic group and c-IAP2 detection status (χ^2^ = 13.41, df = 2, *P* = 0.0012), suggesting that c-IAP2 expression is not uniformly distributed across etiologies but tends to co-occur with the more advanced pulmonary vascular disease (Fig. 7*C*). Given the pilot nature of this analysis and the limited sample size, these findings should be interpreted with caution; nonetheless, they provide preliminary, original, and relevant evidence that c-IAP2 may be implicated in the pathological vascular remodeling that distinguishes established PAH from SchHSD in humans, warranting further investigation in larger cohorts. Echocardiographic analysis comparing RV between *Cdh5creER^t2^;c-IAP1^−/−^:c-IAP2^fl/fl^*sexes after IV/IP Egg exposure supported findings that EC-c-IAP2 depletion seems potentially pathological in a sex-linked manner since females displayed reduced PAT and PAT/PET ratio (Fig. 7 *D* and *F*), increased RVFWTH, with no significant changes in PET, HR, and TAPSE (Fig. 7 *E*, *G*, *H*, and *I*). Given the highly sensitive nature of changes in the host microbiome, the level of circulating Cav-1 and c-IAP2 in plasma may offer broad avenues for optimizing sex-targeted therapies and improving outcomes in pulmonary vascular diseases.

## 3. Discussion

Sch-PAH is the most common form of PAH worldwide (9) and has recently been associated with gut and lung microbiome dysbiosis, as well as significant lung microvascular apoptosis in severely remodeled vessels (4, 7). Dysfunctional gut-lung microbiome composition and cell death are well-known contributors to elevated levels of proinflammatory eATP, which can sustain tissue injury. Functional pathway analysis of lung metadata from the preclinical Sch-PH model indicated a reduction in aerobic respiration compared to controls, often indicative of cellular stress via mitochondrial dysfunction, which is also known to increase ATP secretion. Ex vivo quantification of ATP is often challenging due to its very short lifespan. Once ATP is released from injured cells or activated immune cells in response to pathogens, it can be either rapidly hydrolyzed by ectonucleotidases or activate purinergic receptors such as P2X7R (43, 44). To overcome this limitation, ectonucleotidase inhibition with suramin was performed, revealing elevated lung eATP levels in the IP/IV group. Whereas P2X7R is primarily recognized for its damage-associated role, it has also been implicated in regulating the gut microbiota homeostasis in mice, potentially through its known microbicidal activities (23). Previously, in a cercarial-driven schistosomiasis model using *Swiss* mice, reduced P2X7R expression and function were observed in mesenteric ECs and peritoneal macrophages (16, 45), in contrast to elevated lung EC-P2X7R expression observed in Sch-PH. These data suggest that P2X7R expression may be differentially regulated across organs, vascular beds, and/or disease stages. Specifically, endothelial P2X7R expression was upregulated only in the lung microvasculature, and in vitro blockade of the receptor prevented lung microvascular EC apoptosis, reaffirming its contribution to the microvascular injury observed in Sch-PH.

Although the P2X7R-mediated NLRP3 inflammasome has been implicated in a rat model of PH (18, 46), this pathway was not further evaluated in the Sch-PH model, leaving an important gap for future research. Moreover, both studies used only male rats in a non-pathogen-associated PH model. In contrast to these studies, our work establishes that lung microvascular endothelial P2X7R is a potential therapeutic target in a schistosomiasis-associated preclinical PAH model in both sexes. Specifically, in vitro P2X7R blockade prevented lung microvascular EC apoptosis, while in vivo inhibition was evaluated using BBG. BBG has already demonstrated significant neuroprotective and anti-inflammatory effects, with its clinical use well established in retinal surgeries (47, 48). Systemic therapeutic application remains constrained by poor oral bioavailability, limited absorption, and rapid metabolism, but IP administration resulted in a significant reduction in inflammation with favorable safety (49). While higher concentrations may risk off-target effects, several studies support the notion that doses of 45 or 50 mg/kg constitute an effective, sustainable, and safe therapeutic window in murine models, including those of lung diseases (50). Indeed, P2X7R inhibition-mediated decreased vascular remodeling and RVSP in Sch-PH but not RVH. However, varying the dose regimen or time points of the BBG treatment, evaluating the direct effects of the *S. mansoni* Egg antigens on heart tissue itself, or other sex-linked specific differences, may explain this effect. Regardless, these results strongly indicate that P2X7R may be a potential therapeutic target in Sch-PH pathogenesis.

In terms of molecular mechanism, P2X7R colocalizes with Cav-1 in healthy lung vasculature, where massive ATP efflux occurs in response to inflammation, infection, and tissue injury (38–40). Cav-1 is known as an anti-inflammatory and scaffolding protein crucial for pulmonary microvascular integrity, and its depletion, in part via EV shedding, is closely linked to vascular injury and the expansion of EC apoptosis resistance in experimental, preclinical, and human PAH (7, 11, 51). Recent data indicate that *S*m-p40 promotes Cav-1 phosphorylation and reduction via TLR4/CD14 signaling (7). *S*m-p40 also induced c-IAP2 expression but did not promote cell death or EV shedding. In vitro blocking of P2X7R was sufficient to prevent lung microvascular EC apoptosis and vesicle release, including apoptotic bodies, which contain Cav-1 (11). Cav-1 expression has been implicated in regulating cell death by controlling surviving (41); however, its impact on other IAP family members was previously unknown. Our data display that reduced EC-Cav-1 via EV shedding may also contribute to c-IAP2 depletion in Egg-exposed mice; however, the converse is not fully supported by our new animal model, suggesting that c-IAP2 is a downstream target. Inducible c-IAP2, but not constitutive c-IAP1, was observed to be critical for EC resistance to TNF-α-induced apoptosis (52). In PAH, TNF-α contributes to reduced Cav-1 and BMPRII expression, both essential for lung EC homeostasis. Lung BMPRII expression is also reduced in Sch-PH mice, (7) suggesting a complex link between Cav-1/BMPRII and c-IAP2 signaling in PH. In line with decreased c-IAP2 expression, increased circulating levels of EVs containing c-IAP2 in the plasma of *S. mansoni* Egg-exposed animals suggest that lung endothelial c-IAP2 is shed via a mechanism similar to that of Cav-1 and could serve as a potential biomarker for disease severity.

Previous studies using a global c-IAP1/2 knockout model in a Rosa-creER^T2^ background revealed that systemic c-IAP1 and c-IAP2 deletion led to embryonic death, while conditional deletion of c-IAP2 through *c-IAP2^fl/fl^*contributed to mild lung inflammation (30). c-IAP1 and c-IAP2 were also found to suppress caspase-8-dependent death in vivo, particularly in the intestines and liver, (30) which may contribute to microbiome dysbiosis. Using *c-IAP1^−/−^; c-IAP2^fl/fl^* background under endothelial promoter expression control, we generated, validated, and induced the Sch-PH model. Specifically, homozygous genetic ablation of endothelial c-IAP2 expression resulted in increased lung microvascular remodeling compared with controls. Moreover, the echocardiograph highlighted early pulmonary vascular and RV changes associated with the development of PH or RV overload. Specifically, *S. mansoni* Egg stimulation resulted in a significant reduction in PAT and the PAT/PET ratio, but no changes in PET alone, suggesting an increase in PVR. RVFWTH was significantly increased, confirming RVH. Of note, when sexes were compared, c-IAP2 KO females exhibited higher RVFWTH and unchanged TAPSE, which potentially indicates more pronounced RVH and preserved systolic function, but also showed a lower PAT/PET ratio, reflecting impaired RV-PA coupling. This paradox suggests that although the female RV mounts a stronger adaptation to increased afterload, the pulmonary vasculature may impose a disproportionately higher resistance relative to RV output. These findings indicate that the EC-c-IAP2 expression plays a critical role in the development of Sch-PH. Monitoring trends in these parameters over time in Egg-exposed animals alone or after pharmacological P2X7R inhibition could provide valuable insights into the progression or stabilization of pulmonary and RV function. Together, this study underscores the localized early effects on the RV and pulmonary vasculature in the context of Sch-PH and highlights the relevance of endothelial c-IAP2 in driving these pathological changes.

Yet in terms of the microbiome, it is important to highlight that although our recent work uncovered gut-lung microbiome dysbiosis as a contributing factor to schistosomiasis-associated PH, (7) the underlying mechanisms remain unclear. This study identifies a putative mechanistic link between lung microbiome dysbiosis, tissue injury, and eATP-mediated P2X7R activation, culminating in c-IAP2 suppression and microvascular EC apoptosis. Although many possible mechanistic relationships remain to be investigated, the findings here represent a connection between vascular EC-P2X7R and c-IAP2 in the context of PAH and microbiome dysbiosis. These findings also offer insight into how interorgan interactions influence EC homeostasis in PH, a complex and understudied process. Moreover, the potential sex-specific effects of lung microbiome dysbiosis and EC-c-IAP2 depletion on PAH remain poorly defined, underscoring the need for further investigation. While metagenomic studies continue to expand our understanding of host-microbiome interactions in health and disease, current technical challenges-such as high data variability, limited reproducibility, and insufficient support for cross-model comparisons-highlight the need for supporting more extensive validation within and across systems to support the development of meaningful translational and biotherapeutic approaches.

Beyond the findings discussed above, this study had a few limitations. Echocardiography and IV injections were performed under isoflurane, a volatile anesthetic observed to inhibit TLR4-NLRP3 inflammasome in microglia from diabetic mice (53). Previous studies have also indicated that TLR4 deletion impaired murine hypoxia-induced PH (30). Since P2X7R may activate NLRP3, even though isoflurane’s effects in the lungs are unclear, this potential relationship should be noted. Furthermore, the EC-c-IAP2 model results in systemic c-IAP1 deletion, which did not appear to significantly affect lung vascular homeostasis. Finally, although the consecutive enrollment design and the short interval between blood sample collection and hemodynamic assessment of the patients contribute to minimizing selection bias, the small sample size and the unicentric, tertiary nature of the recruiting center represent relevant limitations, as patients referred to specialized referral centers tend to present more severe disease profiles than those identified at less specialized levels of care. Additionally, the use of a binary detection outcome (detected vs. not detected) rather than continuous c-IAP2 quantification-a consequence of a substantial proportion of samples falling below the assay’s lower limit of detection (<9.4 pg/mL)-precluded more granular between-group comparisons. These aspects underscore the need for caution in interpreting and generalizing the present findings and highlight the importance of multicenter studies with larger sample sizes and optimized quantification strategies for future validation. Despite these limitations, our studies showed that c-IAP2 expression may be critical for lung vascular homeostasis, and that EC-P2X7R overactivation and impaired c-IAP2 expression may contribute to the expansion of the abnormal EC survival phenotype in Sch-PAH (Fig. 7*J*). Further human studies are crucial to determine whether they represent a molecular correlate of established Sch-PAH hemodynamic and remodeled pulmonary vascular disease, positioning them as potentially relevant biomarkers and therapeutic targets in the context of PAH pathogenesis.

## Supplementary Material

Appendix 01 (PDF)

## Data Availability

*Cdh5cre-ER^T2^;cIAP1^−/−^,cIAP2^fl/fl^* model has been generated and validated under disclosure protection by the material transfer agreement #106923 between University of Illinois Chicago and Genentech (https://www.gene.com). Therefore, part of the data is freely available, while the remainder is available upon reasonable request due to privacy agreement restrictions. FASTQ files used in this study are stored in Sequence Read Archive (SRA) under the project PRJNA1473321 (54).
