# Supplementary material for: Endothelial c-IAP2 loss amplifies P2X7 receptor-driven inflammation and worsens schistosomiasis-associated pulmonary hypertension
Source: Proc Natl Acad Sci U S A. 2026 Jun 15;123(25):e2513158123. doi: 10.1073/pnas.2513158123 (PMC13291602; doi:10.1073/pnas.2513158123)
Supplement: Supplementary file 1 — Appendix 01 (PDF) [file pnas.2513158123.sapp.pdf]

Supplementary Material:

Extended material and methods:

Genotyping using Polymerase Chain Reaction: Approximately 1 cm of tail samples were collected from adult animals in a sterile environment and used to perform PCR analysis. PCR was performed using a REDExtract-N-AMP™ Tissue PCR kit protocol (Sigma, Cat #XNAT-100Rxn). For tail tissue digestion, a solution containing 50 μL of extraction solution and 12.5 μL of tissue preparation solution was mixed with individual tail samples and incubated at room temperature for 10 minutes. After the initial incubation, the samples were heated to 95 °C for 3 minutes, then mixed with 50 μL of neutralizing solution B. Depending on the gene of interest, different genotyping protocols were performed. For identification of *Cdhr5*, forward primer (5'-GAT CGC TGC CAG GAT ATA CG-3') and reverse primer (5'-AAT CGC CAT CTT CCA GCA G-3') were used. After mixing each sample, the tubes were transferred to a thermal cycler (Applied Biosystems 2720) for PCR. Initial denaturation occurred at 94 °C for 6 minutes, followed by denaturation at 94 °C for 1 minute. Annealing was at 55 °C for 1 minute, extension at 72 °C for 1 minute and 30 seconds, and a final extension at 72 °C for 5 minutes. 30-35 cycles of denaturation, annealing, and extension were completed before proceeding to the final hold at 4 °C. In parallel, *Birc3* PCR used the same amount of reagents and tissue extracts as Cdhr5, except that three primers were used instead of two, and the annealing and extension cycles were the same. The primers used for *Birc3* were: 5'-GTG GTT TCC AAC GGC TTT G-3'; 5'-AAG TCT AGT CAC AGA GGC TCC AGT-3', and 5'-GAT GGT GGC ACA TGC CTT TAA TCC-3'. The PCR products were then run on a 2-3% agarose gel, and subsequently, the gels were scanned using a Li-Cor Odyssey CLx (Lincoln, NE).

Histological analysis of Pulmonary Vascular Remodeling and immunohistochemistry (IHC): PFA-fixed, paraffin-embedded lung sections (5 μm) were used to evaluate protein expression (primary antibodies: CD31 (R&D systems; AF3628), c-IAP2 (R&D systems; MAB817), and P2X7R (Protein Tech; APR-008) and for histological analysis. Samples were deparaffinized by serial exposure to xylene and ethanol. Then, antigen retrieval was performed for 5-10 min at ~120°C in a pressurized container using 1X sodium citrate as the buffer. For IHC, lung sections were washed with a 0.03% Triton 100x (Sigma-Aldrich, Cat # T8787) buffer diluted in 1X PBS for 2 x 5 minutes. Then, the slides were blocked using 10% goat serum or 5% BSA diluted in PBS (1 hour, room temperature), followed by overnight incubation with the primary antibody at 4°C (in a humidified chamber). After two 5-minute washes, slides were incubated with secondary antibodies, rewashed (3 x 5 minutes), and mounted in mounting media containing DAPI. Then, fluorescent images were collected using an LSM880 confocal microscope (Carl Zeiss MicroImaging, Inc.). Fluorescent images were obtained from randomly selected peripheral lung segments in micro- and macrovessels in each sample to quantify protein expression of interest. Quantification of protein expression per cell was performed using DAPI as a control for cell number. In addition, histological analysis of microvessel area and thickness was quantified in *Masson's Trichrome*-stained sections. After staining, slides containing 1-2 sections were scanned using an Aperio brightfield automated microscope slide scanner (40X; Leica Aperio AT2). Digitalized images were used to determine the microvessel area and thickness (μm) in 10-20 microvessels/animal, i.e., vessels with a diameter smaller than 100 μm, using the ImageScope software 12.4.6 (Leica Biosystems). Briefly, the microvessel wall thickness was measured using the ImageScope ruler tool to determine the length across the thickest part of the vessel segment, defined as the space between the peripheral outer wall of the vasculature and the luminal boundary. The microvessel wall area was calculated by tracing the contours of the peripheral outer wall and the luminal boundary to obtain the total and luminal areas, respectively, and then subtracting the luminal area from the total area

Enzyme-Linked Immunosorbent Assay (ELISA; competitive and multiplex): ELISA was carried out following the protocol from the mouse baculoviral IAP repeat-containing protein 3 (BIRC3; c-IAP2) ELISA Kit (MyBioSource, Cat #MBS7252940). The optimal dilution of plasma samples from control or Egg-exposed

mice (i.e., 1:4) was standardized using 1X DPBS prior to ELISA. All ELISA kit components were brought to room temperature before 100 µL of standards (A-F) and designated samples were added to the appropriate wells in duplicate amounts. Blanks containing only 1x DPBS were also used. 50 µL of conjugate was added to each well, mixed thoroughly, and the plate was then covered and incubated for 1 hour at 37 °C. After 1 hour, the plate was manually washed by aspirating the wells and filling each well entirely with 1x wash solution. Manual washing was completed 5 times before the plate was inverted and blotted on absorbent paper. 50 µL of substrate A and 50 µL of substrate B were added to all wells, incubated for 15 minutes at 37 °C, and then 50 µL of stop solution was added to all wells. The optical density was then measured at 450 nm in a microplate reader (Accuris Smartreader 96). The multiplex ELISA was performed according to the protocol for the mouse premixed multi-analyte Luminex discovery assay kit (R&D Systems, Cat# LXSAMSM). Undiluted and 1:4 diluted whole lung lysate tissue samples were used from control, Egg-exposed, and BBG-treated Egg-exposed mice. All ELISA kit components were brought to room temperature before starting the assay. Briefly, 50 µL of standard or sample was added to the appropriate wells in duplicate, along with 50 µL of microparticle cocktail, and incubated overnight.at 4°C. The next day, the plate was brought to room temperature with shaking on a microplate shaker. Then, the plate was washed 3 times with 100 µL of washing buffer per well. After washing, 50 µL of the biotin-antibody cocktail was added to each well, the plate was covered with foil, and incubation was performed for 1 hour with shaking on a microplate shaker. Washing was repeated after 1 hour, and 50 µL of Streptavidin-PE was added to each well. The plate was covered with foil and incubated for 30 min at room temperature on the microplate shaker. Washing was repeated, and after the final wash, 100 µL of washing buffer was re-added to each well and kept at 4 °C overnight. The next day, the plate was brought to room temperature by shaking on the microplate shaker, then read on the Luminex MAGPIX system, and the results were analyzed using BioTek QuantStudio software and plotted in GraphPad Prism.

Experimental Rodent Echocardiography: At day 0 (D0 - baseline) or day 21 (D21) after IV/IP PBS or Egg exposure, heterozygous and homozygous c-IAP2 mice were anesthetized using inhaled isoflurane (2.5% - 3%) and placed in the supine position on a heating pad. Then, individual animals underwent transthoracic echocardiography using Vevo F2 (VisualSonics Inc., Toronto, ON, Canada) and a UHF57x transducer. A rectal probe continuously monitored body temperature (T = 36.5-37.5 °C). The respiration rate was monitored and controlled by adjusting the depth of anesthesia. RV free wall thickness (RVFWTH) was calculated during end-diastole in the parasternal short-axis mitral valve level, two-dimensional or parasternal long-axis RV outflow tract level, M-mode. In the short-axis view, a pulse-wave Doppler echo was used to record the pulmonary blood outflow at the aortic valve level, measuring pulmonary acceleration time (PAT) and ejection time (PET). Tricuspid annular plane systolic excursion (TAPSE) was measured in 2D M-mode echocardiograms from the apical 4-chamber view, positioning the cursor on the lateral tricuspid annulus near the free RV wall and aligning it as close as possible to the apex of the heart. The severity of pulmonary vascular remodeling (described below) and RVH (described above) was evaluated by histological analysis at the experimental endpoint. Heart rate (HR) was also collected. As a non-invasive imaging method, echocardiography allows real-time assessment of cardiac structure and function.

Human EDTA-plasma samples, ELISA, data preparation and analysis: For the present translational analysis, 13 patients with idiopathic pulmonary arterial hypertension (IPAH), 5 with schistosomiasis-associated pulmonary arterial hypertension (SchPAH), and 12 with hepatosplenic schistosomiasis without pulmonary arterial hypertension (SchHSD) were prospectively and consecutively recruited from patients referred for clinically indicated right heart catheterization at the Pulmonary Hemodynamic Assessment Program of the Federal University of São Paulo (UNIFESP), São Paulo, Brazil. Following written informed consent, venous blood samples were collected immediately before the procedure into EDTA-containing tubes and centrifuged at 1000 ×g for 15 minutes at 2-8°C within 30 minutes of collection. Isolated plasma was aliquoted and stored at -80°C until analysis. BIRC3 plasma concentrations were quantified using a commercially available ELISA kit (MyBioSource, catalog MBS928343), with a detection range of 37.5-2400 pg/mL and a lower limit of detection of < 9.4 pg/mL. The assay demonstrated high specificity for human BIRC3 with no significant cross-reactivity with analogues, and acceptable precision (intra-assay CV < 8%; inter-assay CV < 10%). Optical density (OD) was measured at 450 nm with wavelength correction at 540 nm. The study was approved by the

UNIFESP Institutional Ethics Committee (CEP/UNIFESP CAAE 49617621.1.0000.5505). After obtaining the OD, data were prepared for further statistical analysis. Continuous hemodynamic variables, including mean pulmonary arterial pressure (mPAP), PVR, and pulmonary arterial compliance (PAC), were analyzed. Prior to clustering, variables were standardized using Z-score normalization (mean = 0, standard deviation = 1) to ensure equal contribution to distance calculations. BIRC3 detection status was treated as a binary categorical variable (Yes/No). Samples with an OD value above the blank average were considered positive (Yes). Diagnostic groups were classified as IPAH, SchPAH, and SchHSD. Then, unsupervised hierarchical clustering was performed using Euclidean distance as the similarity metric and Ward's minimum-variance method (Ward.D2) as the agglomeration algorithm. Cluster structure was visually inspected using dendrograms and heatmaps of standardized hemodynamic variables. The number of clusters (k = 3) was defined based on dendrogram structure and silhouette analysis. Cluster validity was assessed using mean silhouette width. The association between clusters and diagnostic groups was assessed as follows: contingency tables were constructed to examine the relationship between hemodynamic cluster assignment and diagnostic group. Pearson's chi-squared test was used to evaluate independence. When expected cell counts were small, Fisher's exact test was applied. An association between clusters and BIRC3 detection was assessed, evaluating the relationship between hemodynamic cluster assignment and BIRC3 detection status using Pearson's chi-squared test and Fisher's exact test when appropriate. The proportions of BIRC3+ patients within each cluster were calculated to determine the direction of association. The association between diagnostic groups and BIRC3 detection was evaluated to determine whether detection differed across categories. All analyses were performed using R (version 4.5.2). Hierarchical clustering was conducted with the stats package, model-based clustering with the mclust package, and silhouette analysis with the cluster package. Given the limited sample size, IPAH and SchPAH were grouped as PAH, and SchHSD as non-PAH to focus the analysis on the presence versus absence of PAH as the primary biological distinction. Larger cohorts will allow stratified analyses by etiology to determine if specific differences exist beyond the shared hemodynamic phenotype.

Reagents and Antibodies: REDExtract-N-AMP™ Tissue PCR kit protocol (Sigma, Cat #XNAT-100Rxn), baculoviral IAP repeat-containing protein 3 (BIRC3; c-IAP2) ELISA Kits (MyBioSource, Cat No. MBS7252940 and Cat No. MBS928343), Mouse Caveolin-1 ELISA kit (Antibodies.com; Cat No. A313603) DPBS, ECL kit (Super Signal West Pico PLUS; REF: 34580; Thermo Scientific), Bio-Rad Protein Assay Kit II (Cat No. 5000002; Bio-Rad), Radioimmunoprecipitation assay (RIPA; Cat No. J63306.AK; Thermo Scientific), APC Annexin V Apoptosis Detection kit with PI (Cat No. 640932; Biolegend), Trypsin-EDTA 1× (Cat No. 15400-054; Gibco), Staurosporine (STS; Cat No. 1285; Tocris), A740003 (Cat. No. 3701, Tocris), TNF-α (Cat No. GF023; Millipore), IFN-Ɣ(Cat No. IF002; Millipore), ATP (Cat No. A2383; Sigma Aldrich), Brilliant Blue G (BBG; P2X7R pharmacological inhibitor; Cat No. BB0770; Sigma), Endothelial basal medium-2 (EBM-2; Cat No. CC-3156; Lonza), MV SingleQuots (Cat No. CC-4147; Lonza), SingleQuots (Cat No. CC-4176; Lonza), Corn oil (Cat No. C8267; Sigma Aldrich), Tamoxifen (Cat No. J63509.03; Thermo Scientific). Rabbit NLRP3 antibody was acquired from Cell Signaling (Cat #1510S). Rabbit polyclonal anti-GAPDH was acquired from Santa Cruz Biotechnology (Santa Cruz, CA, USA). Alexa-Fluor 488 and 555-conjugated goat anti-mouse and anti-rabbit IgG were purchased from Life Technologies (Grand Island, NY, USA). Anti-mouse and anti-rabbit HRP-conjugated IgG were purchased from Cell Signaling Technology (Danvers, MA, USA) or Kierkegaard & Perry Laboratories (Gaithersburg, MD). RIPA buffer, protease and phosphatase inhibitor cocktail, collagenase type I, PFA, sodium citrate, heparin, and sucrose were purchased from SIGMA Chemical Co. (St. Louis, MO, USA). Suramin was purchased from Ambeed (A397022-003). Mounting media with DAPI (VectaShield) was obtained from Vector (Burlingame, CA, USA). The batches 03614, 03577, and 03228 from Sm-p40 were acquired from Biomatik (Wilmington, Delaware, USA). Stock solutions were prepared in 100% dimethyl sulphoxide (DMSO) or sterile PBS and diluted daily in sterile PBS or cell medium for *in vitro* treatments. The highest final concentration of the solvent was 0.1% (v/v) and did not affect the experiments. PCR primers purchased from Integrated DNA Technologies, Inc. (Coralville, Iowa, USA).

Statistics and power analysis: An initial estimate of the number of animals needed per group was calculated based on a Power analysis, which determined the need for n = 6 mice/group to detect statistical differences between groups by ANOVA (an effect size of 25% with a standard deviation of 15% is reached at 80% power and an alpha level of 0.05 with n = 5.26 animals per group; UCLA Dept. of Statistics Power Calculator). A

group size of 6 was found to have statistically significant differences in our previous studies, as determined by ANOVA. Two extra animals/group were included, as we expected to lose preparations due to technical surgical errors (such as a small nick on the lung surface, severed vessels during isolation, anesthesia overdose, or blood clots that limit complete lung perfusion). Data were analyzed using One Codex Cloud Platform and GraphPad Prism v10 (GraphPad, La Jolla, CA, USA). Normally distributed data are presented as the arithmetic mean +/- Standard Error of the Mean. The Shapiro-Wilk test was used to assess normality, and the Brown-Forsythe or F-test was used to assess variance equality. Then, a parametric or nonparametric test was performed accordingly. Parametric statistical analysis was performed using the unpaired Student t-test between two groups, or One-Way or Two-way ANOVA, followed by post hoc analysis (Bonferroni, Dunnett, or Tukey Multiple Comparison tests) between more than two groups. Nonparametric analysis was performed using the Mann-Whitney test. All results were subjected to the ROUT outliers test and were two-sided. $P < 0.05$ was considered statistically significant.

Supplemental Figures and legends:

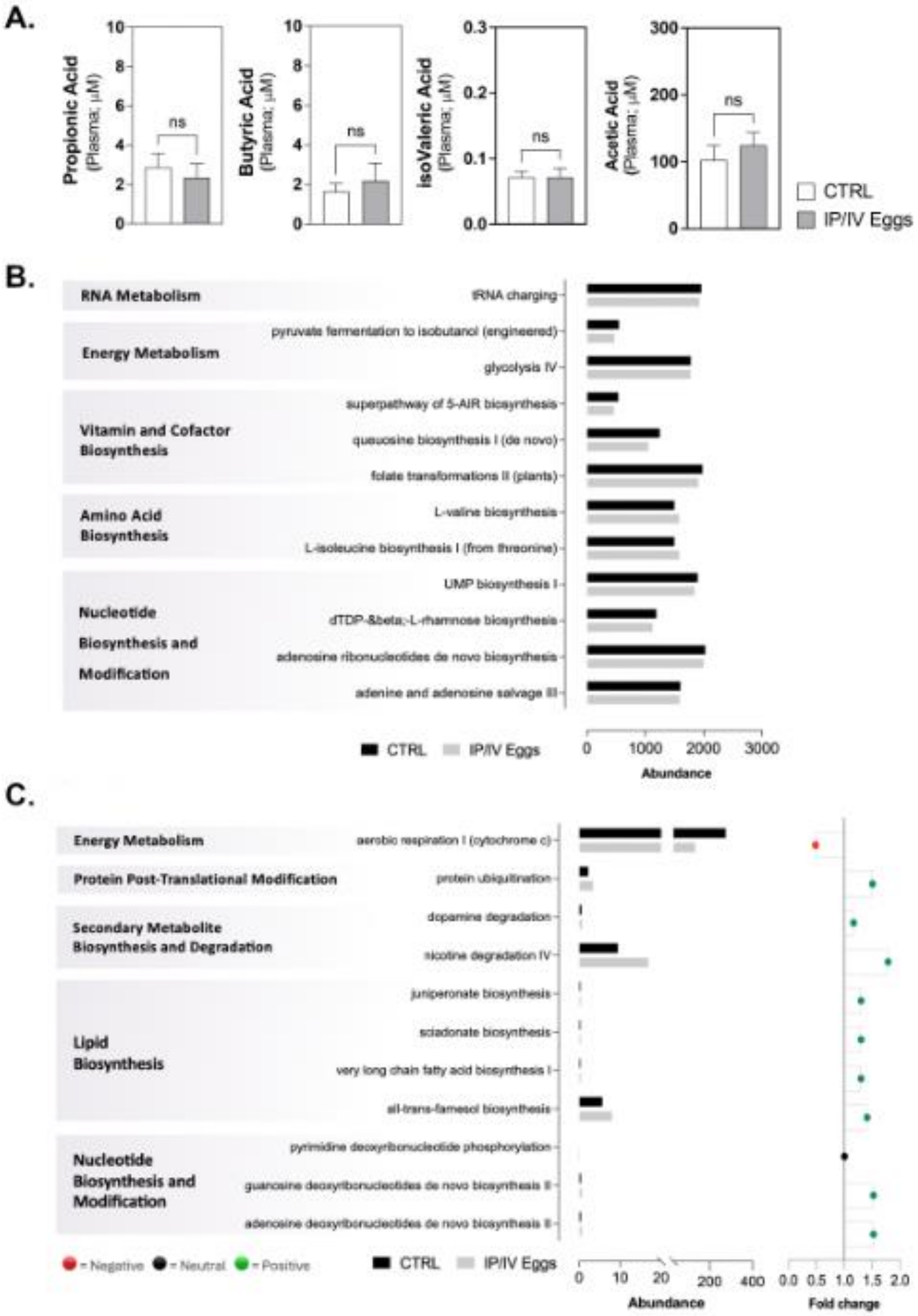

Supplemental Figure 1. Energy metabolism may differ in the preclinical Sch-PH mouse model. Plasma from control and egg-exposed mice was analyzed by liquid chromatography-mass spectrometry to quantify short-chain fatty acid (SCFAs) metabolites: propionic acid, butyric acid, isovaleric acid, and acetic acid (**A**). Functional pathway analysis of the gut (top panel) and lung microbiome (bottom panel) from control and egg-exposed mice (**B-C**). White bars = CTRL, gray bars = IP/IV eggs. Data was analyzed by Student's *t*-test (n = 7-8 animals/group; at least 3-4 mice of each sex, ns = non-significant; *P <0.05; **P<0.01; ***P<0.001; ****P<0.001).

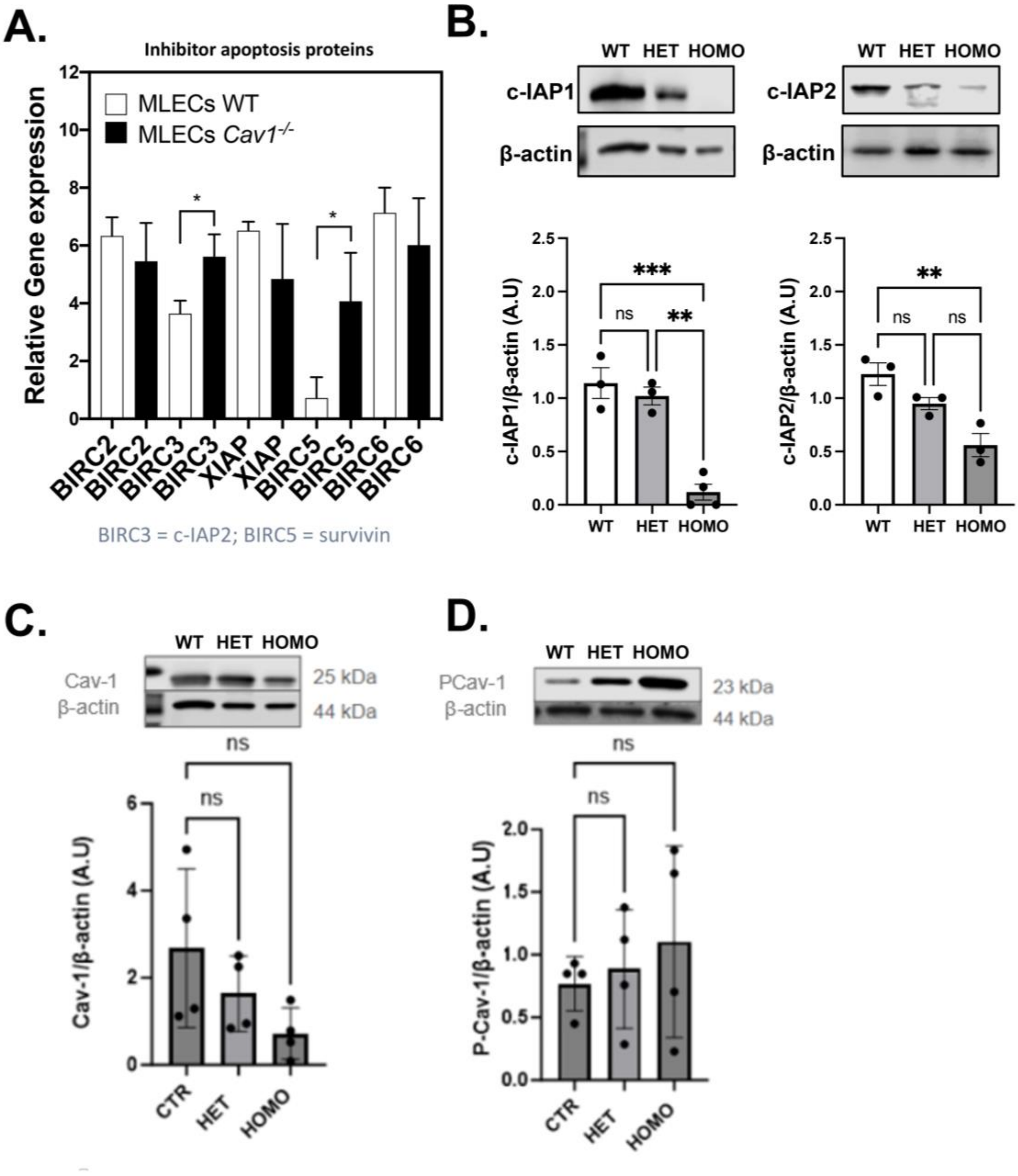

Supplemental Figure 2. c-IAP2 expression may be reliant on Caveolin-1 expression. Relative gene expression of the Inhibitors of Apoptosis family (IAPs) in wild type mouse lung endothelial cells (MLEC's WT) and caveolin-1 knockout mouse lung endothelial cells (MLECs *Cav1*$^{-/-}$; **A**). Western blot of whole lung lysates from wildtype (WT), heterozygous (*Cdh5creER*^{t2}*;c-IAP1*$^{+/-}$*;c-IAP2*$^{+/fl}$), and homozygous c-IAP2 knockout mice (Cdh5cre-ER^{t2};cIAP1$^{-/-}$,cIAP2$^{fl/fl}$) for c-IAP1 and c-IAP2 expression (**B**), Caveolin-1 expression (**C**), and phosphorylated-caveolin-1 expression (**D**). White bars = CTRL, light gray bar = heterozygous (HET), dark gray bar = homozygous (HOMO). Data was analyzed by One-Way ANOVA (n = 3-4 animals/group - at least 1 mouse of each sex; ns = non-significant; **P <0.05; **P<0.01; ***P<0.001; ****P<0.001).

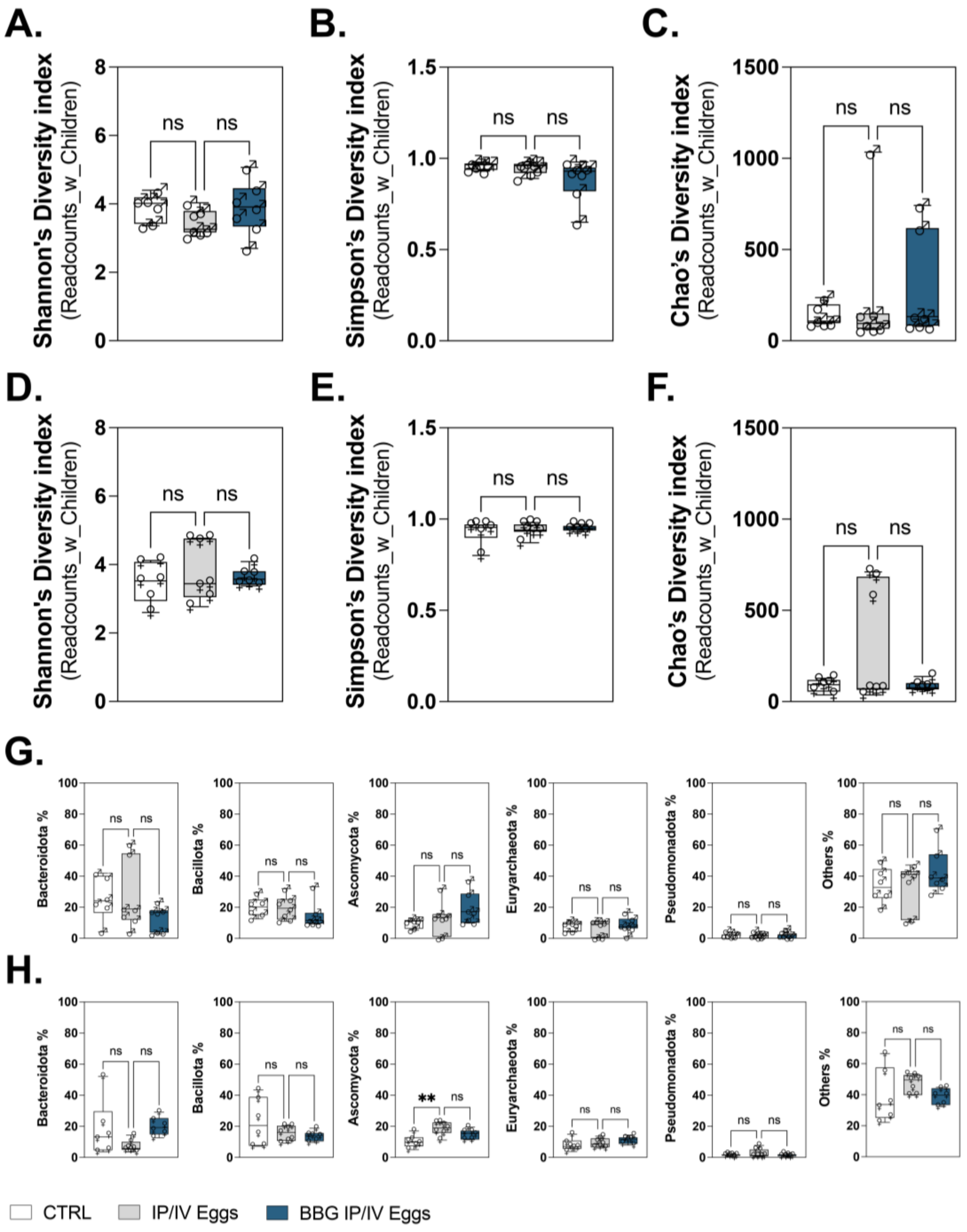

Supplemental Figure 3. Sex differences in CTRL, IP/IV Eggs, and BBG IP/IV Eggs. Lung samples from male **(A -C, G)** and female (D – F, H) CTRL, IP/IV Eggs, and BBG IP/IV mice were used to quantify Shannon's, Simpson's, and Chao's diversity indices via metagenomic analysis **(A-F)**. Phylum percentage for male and female lung samples from metagenomics for Bacteroidota, Bacillota, Ascomycota, Euryachaeota, Pseudomonadota, and others (**G-H**). White bars = CTRL, gray bars = IP/IV Eggs, blue bars = BBG IP/IV Eggs. Data was analyzed by One-Way ANOVA (n = 6-7 animals/group – at least 3 mice of each sex; ns = non-significant; **P <0.05; **P<0.01; ***P<0.001; ****P<0.001).
